# Targeting the PIM protein kinases for the treatment of a T-cell acute lymphoblastic leukemia subset

**DOI:** 10.18632/oncotarget.16320

**Published:** 2017-03-17

**Authors:** Sathish K.R. Padi, Libia A. Luevano, Ningfei An, Ritu Pandey, Neha Singh, Jin H. Song, Jon C. Aster, Xue-Zhong Yu, Shikhar Mehrotra, Andrew S. Kraft

**Affiliations:** ^1^ University of Arizona Cancer Center, University of Arizona, Tucson, AZ, USA; ^2^ Department of Pathology, Pediatric Hematology/Oncology Division, University of Chicago, Chicago, IL, USA; ^3^ Department of Cellular and Molecular Medicine, University of Arizona, Tucson, AZ, USA; ^4^ Department of Pathology, Brigham and Women's Hospital and Harvard Medical School, Boston, MA, USA; ^5^ Department of Microbiology and Immunology, Medical University of South Carolina, Charleston, SC, USA; ^6^ Department of Surgery, Hollings Cancer Center, Medical University of South Carolina, Charleston, SC, USA

**Keywords:** PIM kinase, T-ALL, ETP-ALL, tyrosine kinase inhibitor, ponatinib

## Abstract

New approaches are needed for the treatment of patients with T-cell acute lymphoblastic leukemia (T-ALL) who fail to achieve remission with chemotherapy. Analysis of the effects of pan-PIM protein kinase inhibitors on human T-ALL cell lines demonstrated that the sensitive cell lines expressed higher PIM1 protein kinase levels, whereas T-ALL cell lines with NOTCH mutations tended to have lower levels of PIM1 kinase and were insensitive to these inhibitors. NOTCH-mutant cells selected for resistance to gamma secretase inhibitors developed elevated PIM1 kinase levels and increased sensitivity to PIM inhibitors. Gene profiling using a publically available T-ALL dataset demonstrated overexpression of PIM1 in the majority of early T-cell precursor (ETP)-ALLs and a small subset of non-ETP ALL. While the PIM inhibitors blocked growth, they also stimulated ERK and STAT5 phosphorylation, demonstrating that activation of additional signaling pathways occurs with PIM inhibitor treatment. To block these pathways, Ponatinib, a broadly active tyrosine kinase inhibitor (TKI) used to treat chronic myelogenous leukemia, was added to this PIM-inhibitor regimen. The combination of Ponatinib with a PIM inhibitor resulted in synergistic T-ALL growth inhibition and marked apoptotic cell death. Treatment of mice engrafted with human T-ALL with these two agents significantly decreased the tumor burden and improved the survival of treated mice. This dual therapy has the potential to be developed as a novel approach to treat T-ALL with high PIM expression.

## INTRODUCTION

T-cell acute lymphoblastic leukemia (T-ALL) accounts for 10–15% of pediatric and 25% of adult ALL cases. The disease can be sub-classified into early T-cell precursor ALL (ETP-ALL), cortical, or mature T-ALL based on stage-specific differentiation markers, with ETP-ALLs being defined by the absence of CD4, CD8, and CD1a and frequent expression of one or more myeloid markers [1]. While intensive chemotherapy regimens result in remission in approximately 80% of pediatric and 45% of adult ALL patients, ETP-ALL is associated with a higher rate of relapse and induction failure, with a 10-year overall survival of 19% as compared with 84% for all other T-ALLs [1–3]. T-ALL is driven, at least in part, by the NOTCH signal transduction pathway. Gain-of-function somatic mutations of the gene encoding NOTCH1 are found in 50% of T-ALL patients. In addition, somatic mutations that disrupt the expression of FBXW7, the E3 ligase that mediates the degradation of NOTCH1, results in higher levels of NOTCH1 protein in 15% of T-ALL patients [4]. Downstream targets of NOTCH that contribute to its transforming activity in T-ALL include MYC and the PI3K/AKT pathway, which may be upregulated indirectly through downregulation of PTEN [5, 6]. Patients with ETP-ALL have higher levels of minimal residual disease after induction therapy and thus require more intense and prolonged chemotherapy [3, 7]. Alternative treatment approaches are needed for this disease to obviate intensive chemotherapy and treat resistant disease.

The PIM serine threonine protein kinases were first identified due to their disruption by Moloney murine leukemia virus proviral insertions in murine T-cell lymphomas [8, 9] and play a significant role in hematopoietic malignancies. In normal mouse hematopoiesis, PIM kinases regulate multiple lineages of hematopoietic cells as well as the self-renewal of hematopoietic stem cells (HSCs) [10]. In transgenic mouse models, PIM kinases collaborate with c-MYC, E2A–PBX1, and BCL6 genes to induce B- and T-cell lymphomas [8]. Cytokines such as IL-4 and IL-7 can induce PIM kinases expression in T-cells and promote their growth and survival [11]. PIM protein kinases are overexpressed in a number of hematopoietic malignancies, which is thought to be associated with poor prognosis [12–14]. The crucial role of PIM kinases in hematopoietic tumors has fueled the development of a number of pan-PIM inhibitors that are currently being tested in a Phase I trial in patients with relapsed and/or refractory multiple myeloma and in phase Ib/II trials (CLGH447X2103C; NCT02144038; EudraCT2013-004959-21) for patients with relapsed/refractory acute myeloid leukemia and with high risk myelodysplastic syndrome and myelofibrosis [15, 16].

As PIM kinases contribute to both cell proliferation and survival, they have been implicated in the control of tumor formation [8, 10]. Past research indicated that T-ALL growth can be regulated by PIM kinase inhibitors, and that PIM kinase-associated signal transduction pathways modulated T-ALL growth. Further experiments were performed to determine how Pim inhibitors could be used more effectively to treat this disease [17]. To better understand the role of PIM kinases in T-ALL growth and to assess the potential application of PIM inhibitors in the treatment of T-ALL, we analyzed the effects of two structurally different pan-PIM kinase inhibitors on T-ALL cell lines and carried out a gene profiling analysis using a publically available T-ALL patient dataset Our results demonstrated that PIM kinases are highly overexpressed in the majority of ETP-ALL and in some percentage of non-ETP ALL patient samples. T-ALL cell lines that overexpress PIM1 are sensitive to growth blockade by pan-PIM inhibitors. Furthermore, combining PIM inhibitors with low doses of the tyrosine kinase inhibitor (TKI), ponatinib, induced marked apoptosis in ETP-ALL cell lines in culture, and this combination therapy prolonged the life of mice carrying T-ALL xenografts.

## RESULTS

### Pan-PIM inhibitors can block the growth and inhibit protein synthesis in a subset of T-ALL cell lines

**Figure 1:**
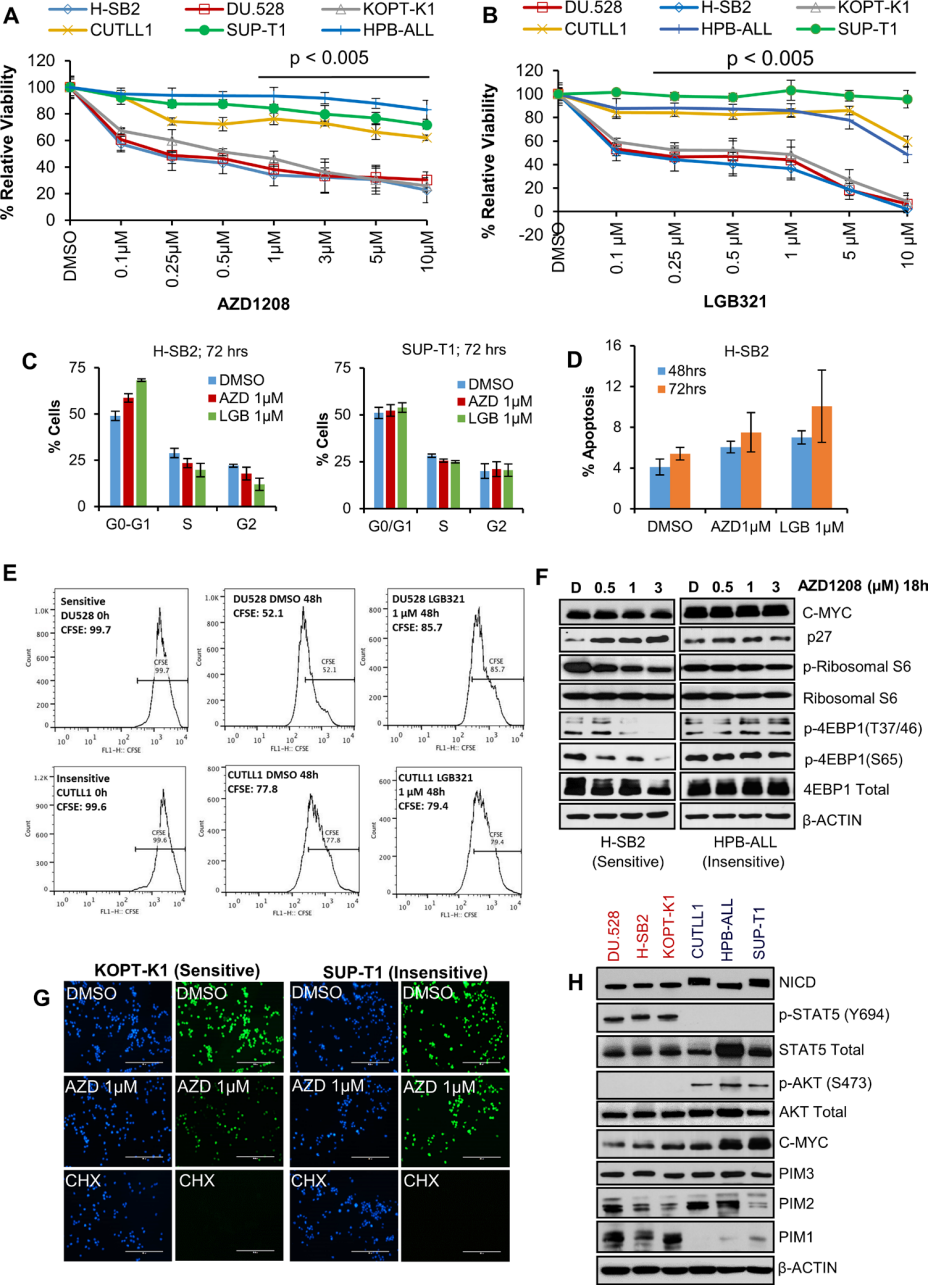
A subset of T-ALL cell lines is sensitive to Pan-PIM kinase inhibitors (**A** and **B**) Human T-ALL cell lines were incubated with the indicated doses of AZD1208 (AZD) or LGB321 (LGB) for 72 h. The percentage of viable cells was measured by XTT assay. The growth of DMSO control for each cell line is considered 100% growth and percentage cell growth for individual treatments is reported relative to the DMSO. Cell growth in PIM inhibitor sensitive (DU.528, H-SB2, and KOPT-K1) cell lines was significantly (*p* < 0.005) inhibited as compared to insensitive cell lines (CUTLL1, SUP-T1, and HPB-ALL). (**C**) H-SB2 and SUP-T1 cells were incubated for 72 h with PIM inhibitors (AZD/LGB) or DMSO. Propidium iodide staining of these cells was followed by cell cycle quantification performed using flow cytometric analysis. (**D**) H-SB2 cells were incubated for 48 h and 72 h with PIM inhibitors (AZD/LGB) or DMSO. Cells were stained with Guava nexin reagent and apoptosis was quantified by flow cytometric analysis. (**E**) DU.528 and CUTLL1 cells were stained with CFSE and incubated for 48h with LGB or DMSO. CFSE fractions at 0 h and 48 h were quantified using flow cytometry analysis. (**F**) H-SB2 and HPB-ALL cells were treated with varying amounts of AZD1208 for 18 h and western blots done with the antibodies listed. (**G**) KOPT-K1 and SUP-T1 cells were treated with DMSO or AZD or Cycloheximide (CHX) for 18 h. Click-iT^®^ HPG Alexa Fluor^®^ 488 Protein Syn-thesis Assay Kit was used to label growing protein chains with fluorochrome seen as green dots. Cell nuclei labeled with nuclear mask blue stain. (**H**) Immunoblot analysis of proteins extracted from PIM inhibitor sensitive and insensitive cell lines using specified antibodies. XTT, cell-cycle, apoptosis and real-time data shown are the average +/− S.D. of three independent experiments. Statistical comparisons performed using an unpaired 2-tailed Student's *t*-test.

To assess the sensitivity of T-ALL cell lines to PIM inhibitors, we compared the effects of two inhibitors with different core chemical structures (AZD1208 and LGB321) on the viability of six human T-ALL cell lines. We found that growth, as determined by an XTT assay, of three cell lines (H-SB2, DU.528, and KOPT-K1) was suppressed in a dose dependent fashion by PIM inhibitors AZD1208 (Figure 1A) and LGB321 (Figure 1B). In contrast, three other cell lines (CUTLL1, HPB-ALL, and SUP-T1) were insensitive to the growth inhibitory effects of these PIM inhibitors.

To further evaluate the mechanism by which PIM inhibitors suppress leukemic cell growth, we investigated the ability of these agents to regulate cell cycle and apoptosis. As shown in Figure 1C, PIM inhibitor treatment of sensitive H-SB2 (but not insensitive SUP-T1) cells partially increased the population of cells in the G0/G1 phase. Because PIM kinase has been shown to regulate the cell cycle by modulating the activity of p27 [17, 18], we evaluated the levels of p27 in PIM inhibitor-sensitive and -insensitive T-ALL cells. Consistent with the cell cycle data, we observed an increase in p27 levels only in PIM inhibitor-sensitive H-SB2 cells (Figure 1F). PIM inhibitor treatment had very minimal effect on cell death at the concentrations utilized (Figure 1D); however PIM inhibitor treatment of sensitive DU.528 and H-SB2 (but not insensitive CUTLL1 and SUP-T1) cells significantly slowed down the cell division, as shown by tracking with the cell-permeant fluorescent dye, carboxyfFluorescein succinimidyl ester (CFSE—high fractions in Figure 1E and Supplementary Figure 1A). The frequency of dividing cells (defined as the CFSE-low fraction) was significantly lower in DU.528 and H-SB2 cells after PIM inhibitor treatment, as compared to DMSO controls. In contrast, there was no significant difference observed in CFSE fractions in CUTLL1 and SUP-T1 cells after PIM inhibitor treatment, as compared to the DMSO control.

Because the PIM protein kinases have been shown to regulate the phosphorylation of 4E-BP1 [19], PRAS40 [20], and also there is evidence that phosphoinositide-3 kinase (PI3K)–AKT and mammalian target of rapamycin (mTOR) pathways are activated in T-ALL [21, 22], we investigated whether PIM inhibitor treatment blocks the mTOR pathway in these leukemic cells. As shown in Figure 1F, the phosphorylation of 4EBP1 and ribosomal S6 was significantly decreased in H-SB2 (a PIM inhibitor-sensitive cell line). However, no inhibition was observed in PIM inhibitor-insensitive HPB-ALL cells. Similarly, PIM inhibitor treatment of sensitive KOPT-K1 (but not SUP-T1, insensitive) cells significantly decreased overall protein synthesis, which was measured by the Click-iT^®^ HPG Alexa Fluor^®^ 488 Protein Synthesis Assay Kit. In comparison, treatment with cycloheximide, a known protein synthesis inhibitor (assay control), significantly reduced protein synthesis in both the sensitive and insensitive cell lines, Figure 1G. PIM inhibitor treatment significantly decreased the phospho-IRS1 (S1101) levels, a known PIM kinase substrate, in all six T-ALL cell lines [23]. Thus, insensitivity to PIM inhibitor treatment did not appear to be driven by drug efflux.

To identify why some of the T-ALL cell lines responded to the PIM inhibitors and the others did not, we evaluated the levels of the PIM isoforms and specific signaling proteins. As shown in Figure 1H, PIM1 protein levels were much higher in the cell lines that were sensitive to PIM inhibitors than those that were insensitive, whereas there was little or no difference in the levels of PIM2 or PIM3. Sensitive cell lines had high levels of phosphorylated (activated) STAT5; in insensitive cell lines, phosphorylated STAT5 was undetectable. In contrast, phosphorylated (activated) AKT levels were low in the sensitive cells lines but high in all three insensitive cell lines. The cells lines also differed in the levels of MYC protein with generally higher levels in the insensitive cell lines. Importantly, the three insensitive cell lines expressed significantly higher levels of activated NOTCH1 protein (NICD1). Together, these experiments demonstrate that PIM inhibitor-sensitive cell lines have higher levels of PIM1 protein and activated STAT, while insensitive cell lines express activated NOTCH and AKT and generally more elevated levels of MYC. The PIM and AKT pathways have overlapping targets and activities [24, 25].

To understand the importance of PIM1 in controlling T-ALL growth, genetic silencing experiments with PIM1-specific siRNAs were performed using H-SB2 cells, a PIM inhibitor-sensitive cell line. As shown in Supplementary Figure 1B and 1D, knockdown of PIM1 demonstrated PIM1 levels were decreased by approximately 50% (siRNA- PIM1; 1 μM, 72 hours), and also caused a decrease in 4EBP1 phosphorylation. A corresponding growth decrease of 25% (Supplementary Figure 1C) was observed when compared to non-silencing control (siRNA-control; 1 μM, 72 hours), suggesting that PIM1 is capable of influencing growth. One reason why the growth effect was not more dramatic is that PIM1 is a member of a family of three PIM kinases. This T-ALL cell lines expresses all three members of the family. Experiments demonstrate increased PIM2 and PIM3 mRNA expression (~ 25%, Supplementary Figure 1E) after knock down of PIM1, suggesting the existence of a compensatory mechanism among the PIM kinases in T-ALL cells, which is similar to what has been reported in other disease models [26, 27].

### Gamma-secretase inhibitor (GSI)-resistant cells contain elevated levels of PIM1 and have increased sensitivity to PIM inhibitors

PIM inhibitor insensitive cell lines (CUTLL1, HPB-ALL, and SUP-T1) are known to have NOTCH mutations that lead to constitutive activation of NOTCH signaling and sensitivity to GSI, which inhibits NOTCH signaling [28, 29]. In contrast, two of the PIM inhibitor-sensitive cell lines (H-SB2 and DU.528) express wild-type NOTCH, and are resistant to GSI-induced growth inhibition [4]. Analysis of The Cancer Genome Atlas (TCGA) data from two independent studies GDS2794 [30] and GDS4303 [31] also demonstrated an inverse relationship between the levels of NOTCH and PIM expression, suggesting that two separate pathways may regulate the growth and transformation of T-ALL. While the third sensitive cell line, KOPT-K1, has activating mutations in NOTCH1 [4], immunoblotting analysis indicated that it shared protein characteristics with the other PIM inhibitor-sensitive cell lines. Notably, KOPT-K1 cells expressed high levels of PIM1 and had an activated STAT signaling pathway.

**Figure 2:**
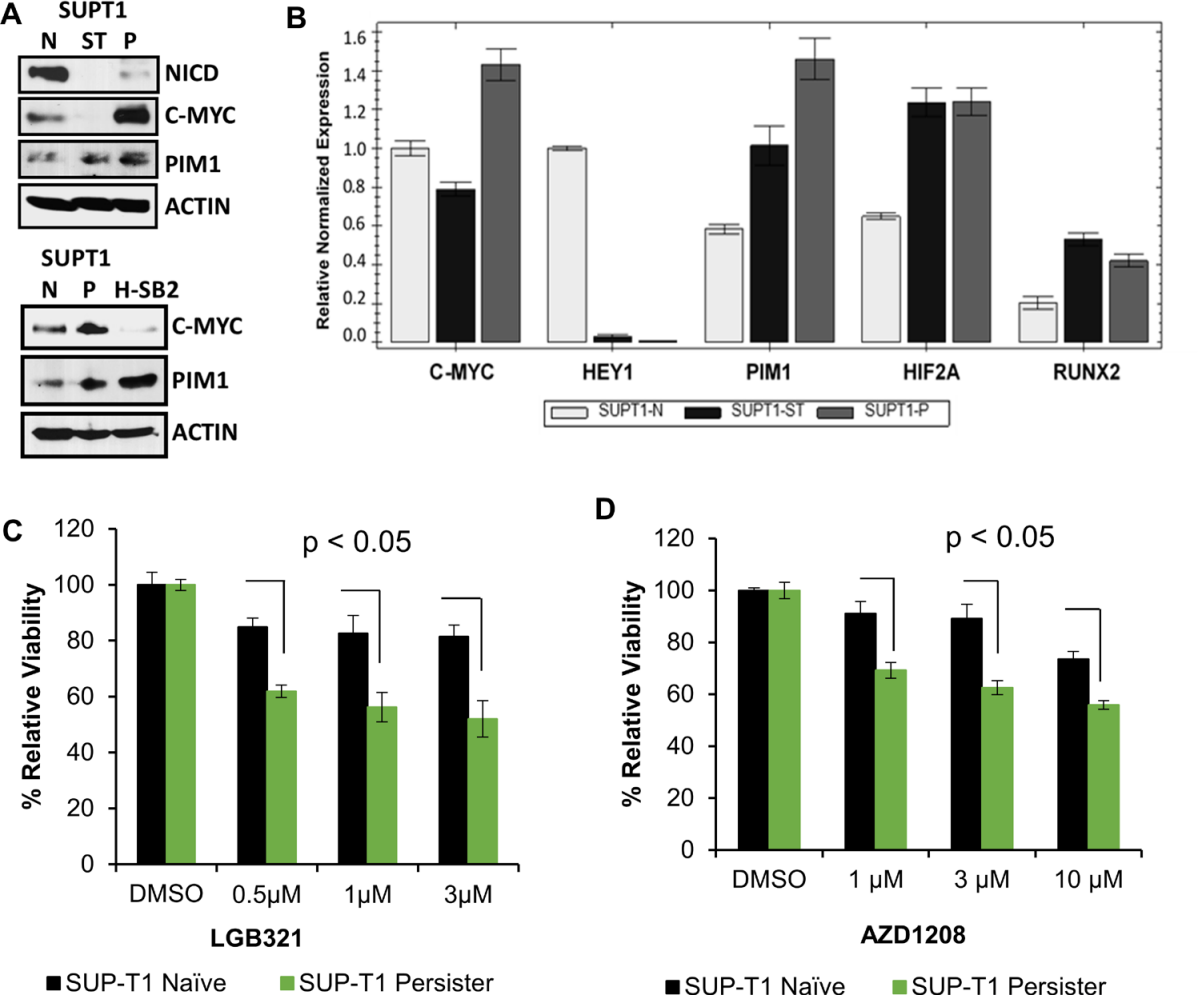
Gamma-secretase inhibitor (GSI) resistant cells overexpress PIM1 and have increased sensitivity to PIM inhibitors (**A**) Immunoblot analysis of activated intracellular NOTCH1 (NICD), c-MYC, and PIM1 levels in SUP-T1 naive, short-term treated (ST) (5 days of 1 μM of the GSI Compound E), persister (P), (7 weeks of 1μM GSI) cells, and H-SB2 cells. (**B**) Total RNA extracted from SUP-T1 naïve (N), short-term treated, and persister cells and the relative mRNA expression of genes shown were analyzed by qRT-PCR. (**C** and **D**) SUP-T1 naïve and persister cells were treated with LGB/AZD for 72 h and *in vitro* cell growth was analyzed using XTT assay. The growth of DMSO control cells is considered 100% and percentage cell growth for individual treatment is reported relative to the DMSO. When compared to naïve cells, SUP-T1 persister cells showed significantly (*p* < 0.05) increased sensitivity to LGB/AZD treatment. XTT and qRT-PCR data shown are the average +/− S.D. of three independent experiments. Statistical comparisons performed using an unpaired 2-tailed Student's *t*-test.

To further characterize the relationship between PIM and NOTCH activity in T-ALL, we utilized a “persister” cell line model [32] in which SUP-T1 cells that are insensitive to PIM inhibitors were cultured continuously with a GSI (Compound E, 1 μM) that is used as a NOTCH-inhibiting therapy for T-ALL [32]. Immunoblotting confirmed that NOTCH1 (NICD1) protein was undetectable in the persister cells (Figure 2A) and that the mRNA level of the NOTCH1 target gene, HEY1, was significantly lower in persister as compared to naïve cells (Figure 2B). As reported previously [32], the persister cells expressed higher MYC mRNA and protein (Figure 2A–2B) than naïve or short-term (ST)-treated SUP-T1 cells. We found that PIM1 protein and mRNA expression (Figure 2A–2B) were higher in the persister cells than the naïve cells. In contrast, PIM2 and PIM3 mRNA levels were not significantly altered, as shown in Supplementary Figure 2. H-SB2 cells were used (Figure 2A, bottom panel) as a positive control for PIM1 protein expression. Similarly, the expression of HIF2A and RUNX2, which are STAT target genes [33], was higher in the persister than the naïve cells (Figure 2B). Furthermore, incubation of the SUP-T1 persister cells with pan-PIM inhibitors produced significant growth inhibition in marked contrast to the resistance of the naïve SUP-T1 cells to these agents (Figure 2C–2D). These data provide further evidence of an association between the levels of PIM kinase activity and the sensitivity of T-ALL cells to PIM inhibitors. The data also suggest that the PIM pathway is activated upon long-term suppression of NOTCH activity in T-ALL cells, thereby providing further credence to the concept that there is an inverse relationship between the activities of NOTCH and PIM kinase.

### PIM1 expression is elevated in the majority of ETP-ALL and in a small percentage of non-ETP ALL patient samples

Microarray-based gene expression profiling studies have shown that T-ALL comprises various molecular subgroups with distinct gene expression signatures. Several important genes, i.e., LYL1, MEF2C, LMO2, and HHEX, show elevated levels of gene expression in ETP-ALL/immature subgroup as compared with other T-ALL subtypes [1, 34]. Currently, ETP-ALL is defined by an immunoprofile that includes absence of CD1a, CD4 and CD8, low CD5 expression, and expression of one or more myeloid or stem cell antigens [1]. As shown in Supplementary Table 1, the immunophenotype of all the insensitive cell lines (CUTTL1, SUP-T1 and HPB-ALL) was consistent with a non-ETP-ALL phenotype (CD4^+^/CD8^+^ and TdT^+^). In contrast, H-SB2 and DU.528, which are PIM inhibitor sensitive cells, had an ETP-ALL-like phenotype (CD4^−^/CD8^−^ and TdT^−^). However, interestingly the immunophenotype of the KOPT-K1 cells, which also were sensitive to PIM inhibitors and had increased PIM1 levels, was similar to a non-ETP-ALL phenotype [1, 4, 35, 36]. This result is consistent with the concept that immunophenotyping may not rigorously define ETP-ALL and that there may be some cases that represent an “intermediate” category of T-ALL with a non-ETP phenotype but elevated PIM1 levels.

**Figure 3:**
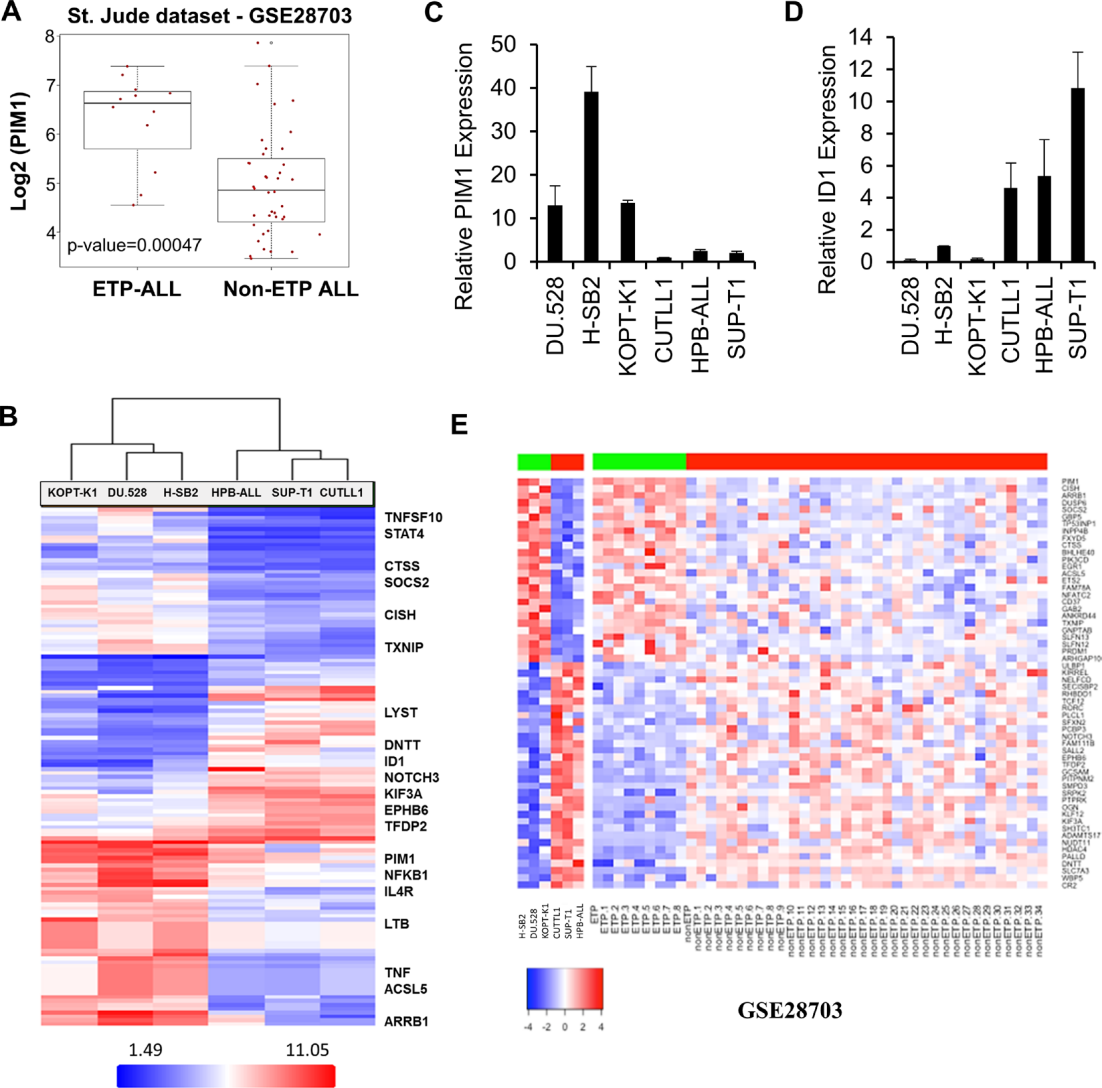
Overexpression of PIM1 in majority of ETP-ALL and a small percentage of Non-ETP ALL patient samples (**A**) Box plot detailing significantly high PIM1 mRNA expression (*p*-value = 0.00047) in ETP-ALL (*n* = 12) as compared to non-ETP ALL (*n* = 40) pediatric patient samples in GSE28703 (St. Jude dataset). (**B**) Heat map of top 135 genes that significantly differentiate (Fold Change (linear) < − 3 or > + 3 and ANOVA *p*-value (Condition pair) < 0.05.) PIM inhibitor sensitive cells from PIM inhibitor insensitive cells. Genes in the heat map are shown in rows and each individual T-ALL cell line is shown in one column. Expression levels are visualized as color-coded with red indicating higher levels and blue lower levels of gene expression. (**C** and **D**) Total RNA was extracted from 6 T-ALL cell lines and the relative mRNA expression of PIM1 and ID1 were analyzed by quantitative real-time polymerase chain reaction. (**E**) Heat map of significantly differential 58-gene signature for both T-ALL cell line data and patient samples from the St. Jude dataset (ETP-ALL; *n* = 9, high PIM1 and Non-ETP ALL; *n* = 35, low PIM1), *p*-value of ≤ 0.05. Genes in the heat map are shown in rows and each individual T-ALL cell line or leukemia patient sample is shown in one column. Expression levels are visualized as color-coded with red indicating higher levels and blue lower levels of gene expression.

To explore the potential association of elevated PIM1 expression with ETP-ALL or immature T-ALL, we analyzed a publically available gene expression dataset GSE28703 [34] containing 52 T-ALL pediatric patient samples. The results demonstrate that PIM1 is highly expressed in 75% (9/12) of patients with ETP-ALL and in 13% (5/40) of non-ETP ALL (*p* = 0.00047; Figure 3A). The classification of T-ALL samples in this cohort was taken as provided.

Microarray profiling was carried out on six T-ALL cell lines using an Affymetrix Gene Chip (HTA 2.0 Array). As shown in Figure 3B, array results were consistent with the data obtained by Western blotting (Figure 1H). The list of the top 135 genes that significantly differentiate sensitive cells from insensitive cells are provided in Supplementary Table 2. PIM1 mRNA expression was significantly higher in the PIM inhibitor-sensitive cell lines (H-SB2, DU.528, and KOPT-K1) as compared to the PIM inhibitor-insensitive cell lines (CUTLL1, HPB-ALL, and SUP-T1). Although there was some variability, PIM2 and PIM3 mRNA levels did not distinguish sensitive from insensitive cell lines (Supplementary Figure 3A–3B). Sensitive cells contained elevated transcription levels of proteins involved in the JAK/STAT (CISH, STAT4, SOCS2, JAK3, and HIF2A) and NFkB pathways. The insensitive cell lines were found to have elevated transcription levels of proteins involved in NOTCH signal transduction pathways (TdT or DNTT, ID1, HDAC4, NOTCH3, HES1, and HEY1). Microarray analysis was validated using qRT-PCR; there were significant differences in mRNA expression of PIM1, CISH, HIF2A, ID1, and HEY1 (Figure 3C–3D and Supplementary Figure 3C–3E) between PIM inhibitor sensitive and insensitive cell lines. Collectively, these studies demonstrate that these T-ALL cell lines could be grouped into PIM inhibitor-sensitive and -insensitive subgroups based on the mRNA and protein levels of specific genes in distinct pathways.

To obtain a more complete understanding of the genotypes associated with PIM1 expression that may contribute to a sensitivity to PIM kinase inhibitors, gene signatures were generated by further analysis of ETP and non-ETP cases identified, respectively as having high PIM1 mRNA expression (*n* = 9) versus low PIM1 mRNA expression (*n* = 35) in St. Jude data set, GSE28703 [34]. The analysis was carried out independently using Bioconductor LIMMA modules and R statistical tools [37, 38]. This led to the identification of 58 genes (Figure 3E) that were significantly different (26 upregulated; 32 downregulated) in the PIM1 overexpressing and underexpressing T-ALL samples [34]. Using an adjusted *p*-value of ≤ 0.05 as the cutoff for significance, these genes also could differentiate between the PIM inhibitor-sensitive and -insensitive T-ALL cell lines using the mRNA expression profiling dataset described earlier. A correlation analysis of PIM inhibitor-sensitive and insensitive T-ALL cell lines with St. Jude T-ALL patient dataset (GSE28703) [34] showed that the genes expressed in the PIM inhibitor-sensitive cells are highly correlated with the results obtained from ETP-ALL patient samples than the more mature T-ALL samples (Supplementary Figure 3F). Collectively, these studies demonstrate that T-ALL cell lines could be grouped into PIM inhibitor-sensitive and -insensitive subgroups based on the mRNA and protein levels of specific genes in distinct pathways. A Cytoscape [39] network of significantly enriched candidate pathways and their associated genes generated from Reactome [40] and MSigDB [41, 42] is shown in Supplementary Figure 3G. Genes associated with increased PIM1 expression (shown in red) that were upregulated in ETP-ALL included molecules involved in cytokine immune signaling, Ras signaling, and IL-2/STAT5-activated genes. Genes shown in green were underexpressed in ETP-ALL patients or PIM inhibitor-sensitive cells, and the majority of these genes belong to the NOTCH signaling pathway. A list of enriched pathways and their associated genes is provided in Supplementary Table 3.

### Synergistic effect of pan-PIM inhibitors with TKIs in a subset of T-ALL cells

**Figure 4:**
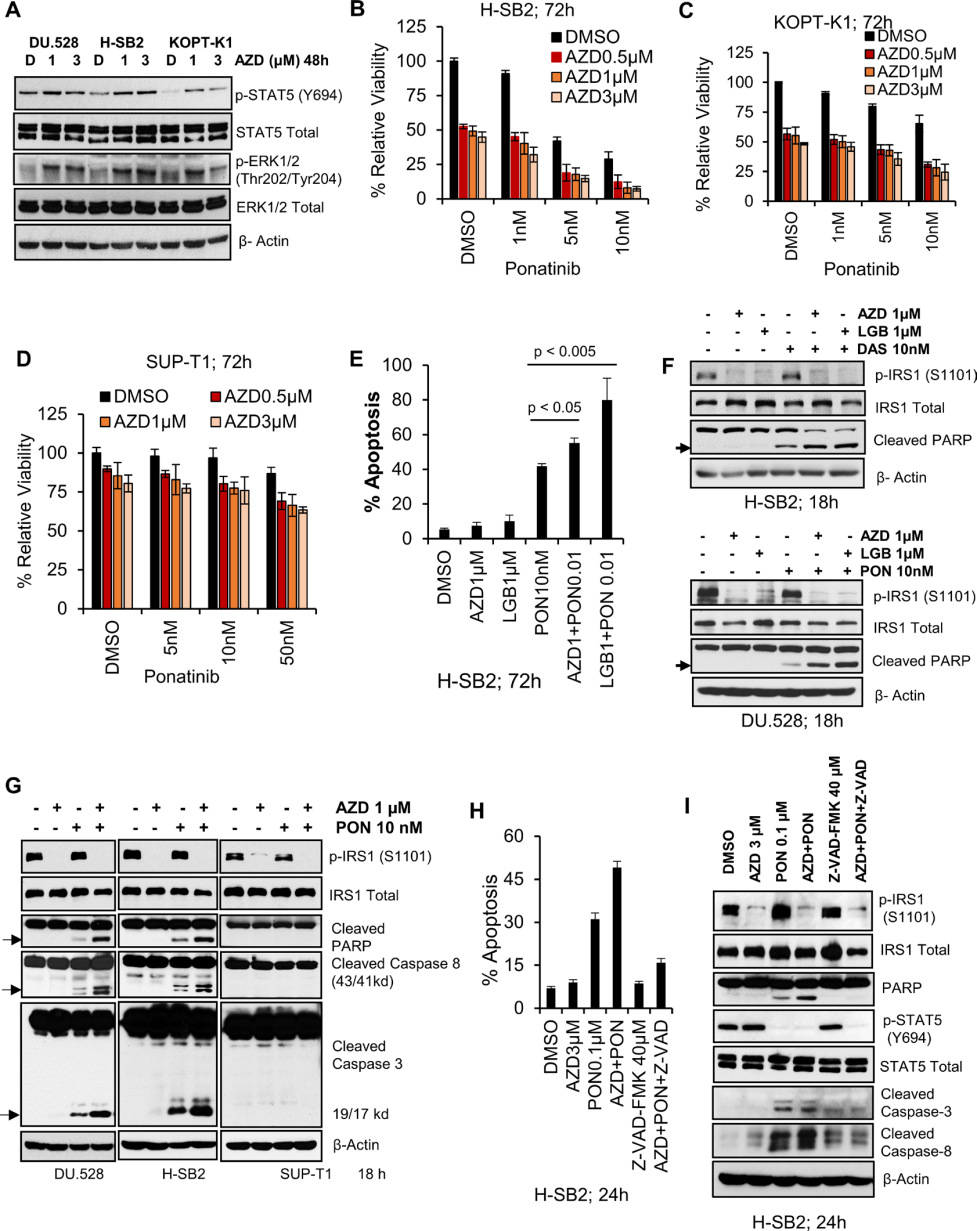
Pan-PIM inhibitors enhance apoptosis induced by tyrosine kinase inhibitors (**A**) PIM inhibitor sensitive cell lines (H-SB2, DU.528, and KOPT-K1) were incubated with AZD for 48 h and immunoblotted with specified antibodies. (**B**–**D**) H-SB2, KOPT-K1 and SUP-T1 cell lines were incubated with AZD either alone or in combination with Ponatinib at indicated doses for 72 h and cell viability was measured by XTT. The growth of DMSO control cells is considered 100% and percentage cell growth for individual treatment is reported relative to the DMSO control cells. (**E**) H-SB2 cells were incubated with AZD or LGB either alone or in combination with 10 nM Ponatinib for 72 h. Apoptosis was measured using Guava nexin assay followed by flow cytometry. (**F**) PIM inhibitor sensitive cells lines (H-SB2 and DU.528) were treated with DMSO, AZD/LGB alone or in combination with Ponatinib (PON) / Dasatinib (DAS) for 18 h and cell lysates were immunoblotted with specified antibodies. (**G**) DU.528, H-SB2, and SUP-T1 cells were treated with DMSO, AZD alone or in combination with PON for 18 h and cell lysates were immunoblotted with specified antibodies. (**H**) H-SB2 cells were either pretreated with DMSO or 40 μM Z-VAD-FMK (a pan-caspase inhibitor) for 2 h, followed by the addition of AZD, PON or the combination for 24 h. The percentage of apoptotic cells was quantified using the Guava nexin assay followed by flow cytometry. The level of apoptosis in AZD plus PON versus PON treatment significantly differed (*p*-value < 0.05). (**I**) H-SB2 cells were treated similarly to (H) and cell lysates were immunoblotted with the specified antibodies. The XTT, apoptosis and flow cytometry data shown is the average +/− S.D. of three independent experiments. Statistical comparisons performed using an unpaired 2-tailed Student's *t*-test.

Incubation with PIM inhibitors blocks growth of sensitive T-ALL cell lines, but does not induce cell death (Figure 1A–1D). When sensitive T-ALL cells were treated for a short period with PIM inhibitors (Figure 4A), phosphorylation of STAT5 and ERK1/2 was increased, suggesting that signaling pathways were being activated in response to these inhibitors. Previously, we have shown that inhibition of the PIM kinase pathway is associated with increased ERK1/2 phosphorylation and that combining PIM and MEK inhibitors synergistically decrease the leukemia burden [17]. Hormone-binding receptor tyrosine kinases in T-cells activate the lymphocyte-specific tyrosine kinase, LCK, which plays a crucial role in T-cell development and is tyrosine kinase (TK) that is predominantly enriched in the T-ALL patient-derived xenografts [43]. Additionally, whole-genome and transcriptome sequencing of high risk T-ALL subsets (including ETP-ALL [34, 44] and Philadelphia chromosome-like ALL [45, 46]) reveal activating mutations that result in constitutively activated TK. Considering the important role of LCK in T-ALL and its ability to activate STAT and other downstream signaling pathways [47, 48], we evaluated the activity of a TKI, ponatinib, alone and in combination with PIM inhibitors. Ponatinib is a multi-targeted TKI approved for human use in chronic myeloid leukemia (CML) [49] that has been shown to have broad activity against SRC family members including LCK [50]. As reported earlier [51], ponatinib treatment alone induced a marked inhibition of STAT and ERK phosphorylation, while it had no effect on p-IRS1, a substrate of PIM kinase [23] (Supplementary Figure 4A). Combination therapy with ponatinib and AZD1208 caused a significant decrease in proliferation of H-SB2 and KOPT-K1 cells, but not SUP-T1 cells (Figure 4B–4D). Also the combination treatment significantly increased apoptotic cell death, (Figure 4E) and induced G0-G1 cell cycle arrest (data not shown) in H-SB2 cells. Notably, Combosyn analysis [52] demonstrated that AZD1208 and ponatinib combination was highly synergistic in killing H-SB2 and KOPT-K1 cell lines. As shown in the Supplementary Figure 4B–4C, the combination index (CI) values were less than 1 for various AZD1208 and ponatinib combination doses. Highly similar results were obtained using another PIM inhibitor sensitive cell line, DU.528 (but not using insensitive CUTLL1) (Supplementary Figure 4D–4E). In contrast, SUP-T1 cells required significantly higher concentrations of ponatinib (IC_50_ ~500 nM; Supplementary Figure 4F–4G) to induce cell killing. This combined effect was not limited to ponatinib but also was seen with another broad-spectrum TKI, dasatinib, which has been approved for treatment of CML [53]. As shown in Supplementary Figure 5A–5B, combination therapy with dasatinib and PIM inhibitors (AZD1208 or LGB321) caused a significant decrease in proliferation of KOPT-K1 cells, but not SUP-T1 cells.

The increase in T-ALL apoptotic cell death after combination therapy was associated with a marked increase in PARP cleavage. In the PIM inhibitor-sensitive cell lines (H-SB2 and DU.528), PARP cleavage was seen after treatment with the ponatinib or dasatinib, but was significantly increased by the concomitant application of a PIM inhibitors (AZD1208 or LGB321), as shown in Figure 4F. In H-SB2 and DU.528 cell lines, the synergistic PARP cleavage was paralleled by cleavage of caspase 3 and 8. Identical doses of these agents did not cause PARP and caspase cleavage in PIM inhibitor-insensitive SUP-T1 cells (Figure 4G). The addition of the pan-caspase inhibitor z-VAD-FMK was capable of blocking the induction of apoptosis caused by the combination treatment in H-SB2 cells, as shown in Figure 4H and Supplementary Figure 6A. Immunoblot analysis showed that addition of a pan-caspase inhibitor prior to the drug treatment significantly blocked both PARP and caspase cleavage (Figure 4I). The caspase-8 inhibitor partially rescued cells from apoptosis induced by the combination treatment (Supplementary Figure 6B), suggesting the importance of caspases in the induction of cell death.

**Figure 5:**
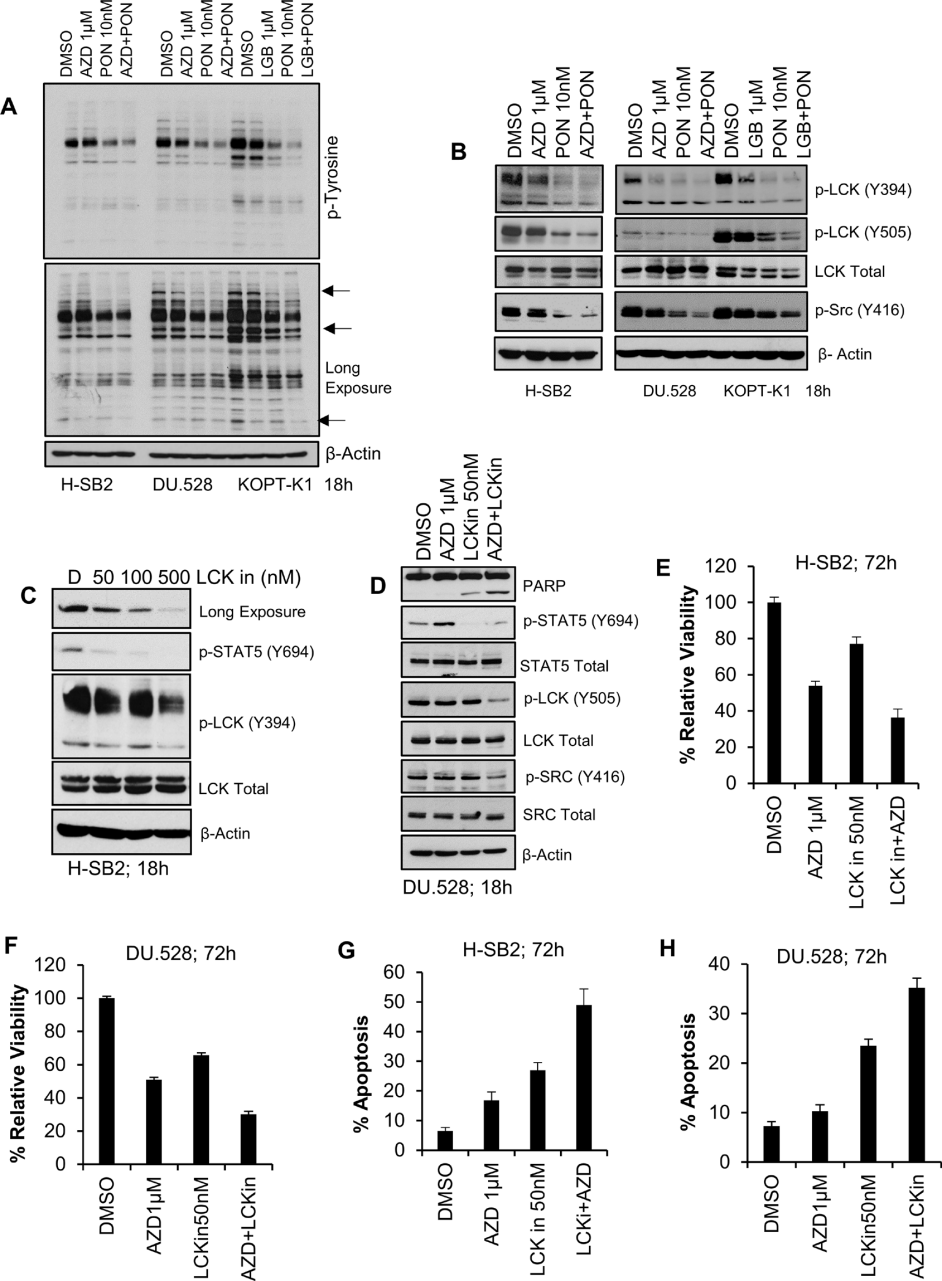
Synergistic effect of pan-PIM inhibitor(s) with Ponatinib or LCK inhibitor (LCKin) in T-ALL cell lines (**A**, **B**) PIM inhibitor sensitive cells lines (H-SB2, DU.528, and KOPT-K1) were treated with DMSO, or AZD alone or in combination with Ponatinib for 18 h and cell lysates were immunoblotted with anti phospho-tyrosine antibody (A) or the specified antibodies (B). (**C**) H-SB2 cells were treated with LCKin at the indicated concentrations for 18 h. Cell lysates were immunoblotted with specified antibodies. (**D**) DU.528 cells were treated with DMSO or AZD alone or in combination with LCKin for 18 h and cell lysates were immunoblotted with specified antibodies. (**E**, **F**) H-SB2 and DU.528 cells were treated with DMSO, AZD, LCKin either alone or in combination for 72 h. The percent viability was determined by XTT assay. The growth of DMSO control cells was considered 100% and percent cell growth after individual treatment is reported relative to the DMSO. (**G**, **H**) H-SB2 and DU.528 cells were treated with DMSO, AZD either alone or in combination with LCKin for 72 h. The percent of cells undergoing apoptosis was analyzed by the Guava nexin assay followed by flow cytometry. XTT and apoptosis data shown are the average +/− S.D. of three independent experiments.

We have previously demonstrated that PIM inhibitors can decrease the protein levels of multiple cell surface receptor TKs [54]. To examine the possibility that PIM inhibitor treatment blocks tyrosine phosphorylation in these T-ALL cells, whole cell anti-phosphotyrosine immunoblotting was carried out. Ponatinib decreased the levels of total cellular tyrosine phosphorylated proteins, and the addition of a PIM inhibitor further enhanced this effect in sensitive cell lines (Figure 5A). It has been demonstrated that in these T-ALL cells, both SRC and LCK are activated downstream of cell surface receptors, and are known to activate STAT5 signaling [48]. Immunoblotting demonstrated that the combination of AZD1208/LGB321 and ponatinib produced significantly greater inhibition of SRC/LCK phosphorylation in sensitive cell lines (Figure 5B) but not in insensitive SUP-T1 cells (Supplementary Figure 6C and 6D). Also, the ponatinib / AZD1208 combination significantly inhibited p-S6, and p-4E-BP1 levels in the sensitive cell line, H-SB2, but not the insensitive, SUP-T1, cell line (Supplementary Figure 6E). To further determine the importance of LCK in T-ALL, the specific LCK inhibitor [55] was used in combination with the PIM inhibitor, AZD1208. LCK inhibitor alone produced significant inhibition of phospho-STAT5 (Y694) (Figure 5C–5D). When LCK inhibitor (50 nM) was combined with AZD1208 (1 μM), we also observed synergistic inhibition of cell growth (Figure 5E–5F) and induction of apoptosis (Figure 5G–5H) in PIM inhibitor sensitive H-SB2 (CI = 0.68544) and DU.528 (CI = 0.68285) cells, suggesting the importance of this tyrosine kinase in T-ALL growth. These results can explain in part the ability of PIM inhibitors to synergize with broad-spectrum TKIs.

### *In vivo* sensitivity of H-SB2, an ETP-ALL cell line, to AZD1208 and ponatinib combination treatment

**Figure 6:**
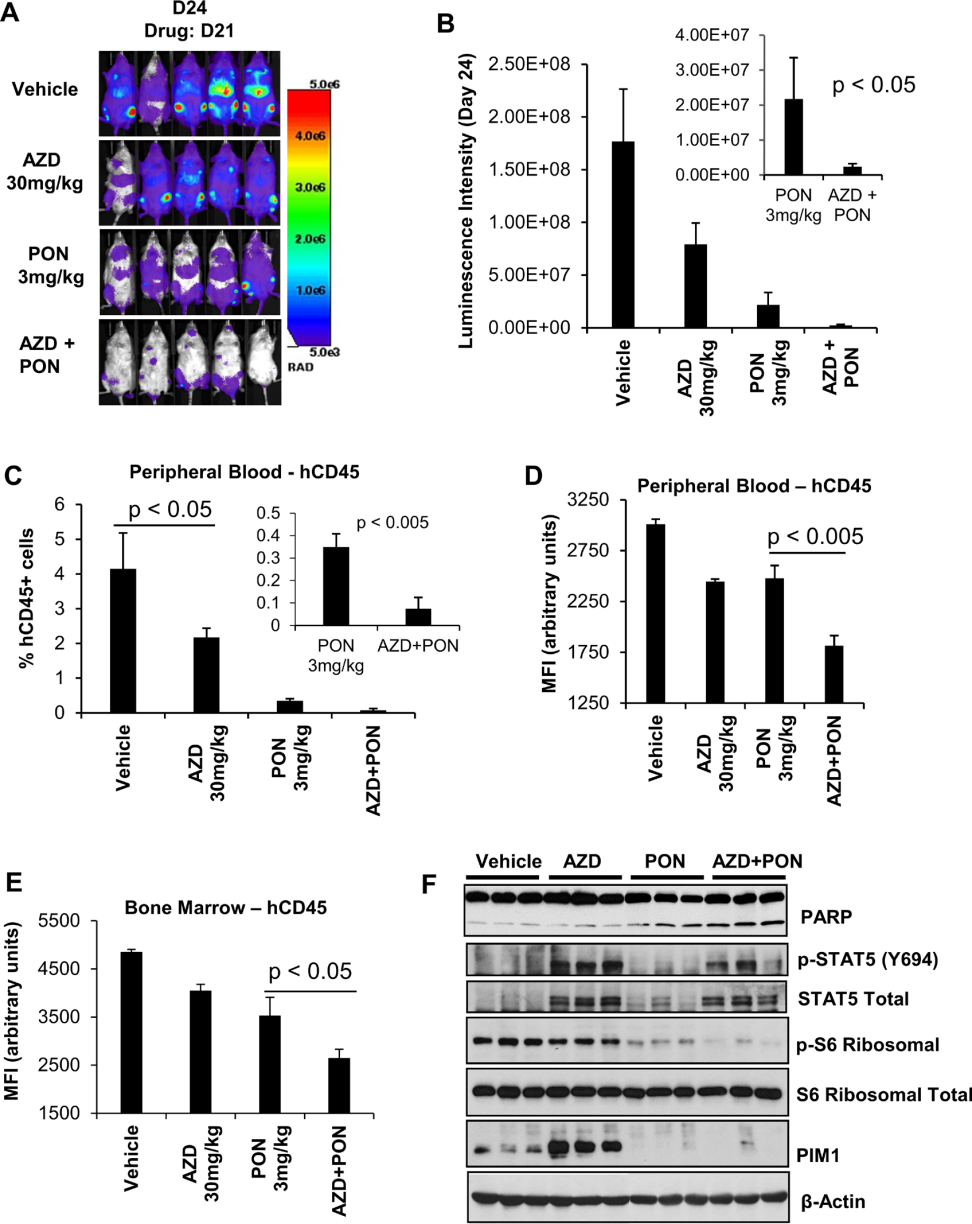
The *in-vivo* sensitivity of H-SB2, an ETP-ALL cell line to AZD1208 (AZD) and Ponatinib (PON) combination treatment (**A** and **B**) Twenty NSG mice that had received sublethal irradiation (2.5 Gy) were injected intravenously with (200,000 cells/100 μL PBS) H-SB2-luc cells by tail vein. On day 3 after injection the 20 mice were randomly assigned to daily treatment by oral gavage with vehicle, AZD 30 mg/kg, PON 3 mg/kg, or the combination for 3 weeks. The tumor burden was assessed by bioluminescence imaging after 3 weeks of treatment. Luminescence intensity is quantified by using AMIView software. The data shown is the average +/− the standard error of the mean (SEM) from five mice per treatment. (**C** and **D**) Flow cytometric analysis of surface hCD45 expression. The percent of hCD45+ cells and median fluorescence intensity (MFI) using flow cytometry in mouse peripheral blood (PB) samples collected from all treatment groups at the necropsy is shown. A significant decrease in the percentage of hCD45+ and MFI of hCD45+ expression is seen in the PB (*p* < 0.005) harvested from AZD plus PON combination therapy versus PON treatment alone. (**E**) Measurement of hCD45+ cell MFI using flow cytometry. Mouse bone marrow (BM) samples were collected from all treatment groups at the necropsy. A significant decrease in the MFI of hCD45+ cells was seen in BM samples (*p* < 0.05) harvested from AZD plus PON combination therapy versus PON treatment alone. MFI and % hCD45+ data shown are the average +/− S.D. from five samples per treatment. Statistical comparisons performed using an unpaired 2-tailed Student's *t* test. (**F**) Immunoblot analysis of bone marrow cells harvested from vehicle, AZD 30 mg/kg, PON 3 mg/kg, or combination using specified antibodies. Each lane on the western blot represents an individual animal tumor.

To evaluate the ability of a TKI plus PIM inhibitor treatment to block tumor growth of ETP-ALL *in vivo*, H-SB2 cells stably expressing luciferase (H-SB2-luc; 200,000 cells/100 μL PBS) were injected intravenously into sub-lethally irradiated (2.5 Gy) NOD/SCID IL-2Rγ−/− (NSG) mice. After three days, the mice were treated with AZD1208 30 mg/kg [56] or ponatinib 3 mg/kg [49], the combination of drugs, or vehicle once daily by oral gavage for a duration of 3 weeks. Combination treatment is non-toxic to the mice in that it did not cause any significant change to the body weight (Supplementary Figure 7A). Tumor growth was monitored at the indicated time points by bioluminescent/fluorescent imaging, as described in Methods. As shown in Figure 6A–6B, when compared to ponatinib alone, combination treatment significantly decreased tumor burden (*p*-value < 0.05). Consistent with the bioluminescence imaging results, flow cytometric analysis using anti-human CD45 antibody to identify leukemic cells [57] demonstrated significantly lower surface hCD45 expression in the peripheral blood (PB, *p*-value < 0.005) and bone marrow (BM, *p*-value < 0.05) samples collected from the AZD1208 plus ponatinib-treated mice when compared to the mice treated with ponatinib alone (Figure 6C–6E, and Supplementary Figure 7B). Western blot analysis of BM cells (Figure 6F) showed increased PARP cleavage and a significant decrease in ribosomal phospho-S6 levels in mice treated with AZD1208 plus ponatinib compared to the individual inhibitor treatments. As expected from cell culture experiments, mice treated with AZD1208 had activated STAT signaling; this activation was significantly inhibited in mice with combination treatment. Following treatment with AZD1208, induction of PIM1 protein levels was increased, as previously reported with other PIM inhibitors [58]. These results support dual inhibitor treatment for a specific subset of T-ALL tumors expressing PIM1.

**Figure 7:**
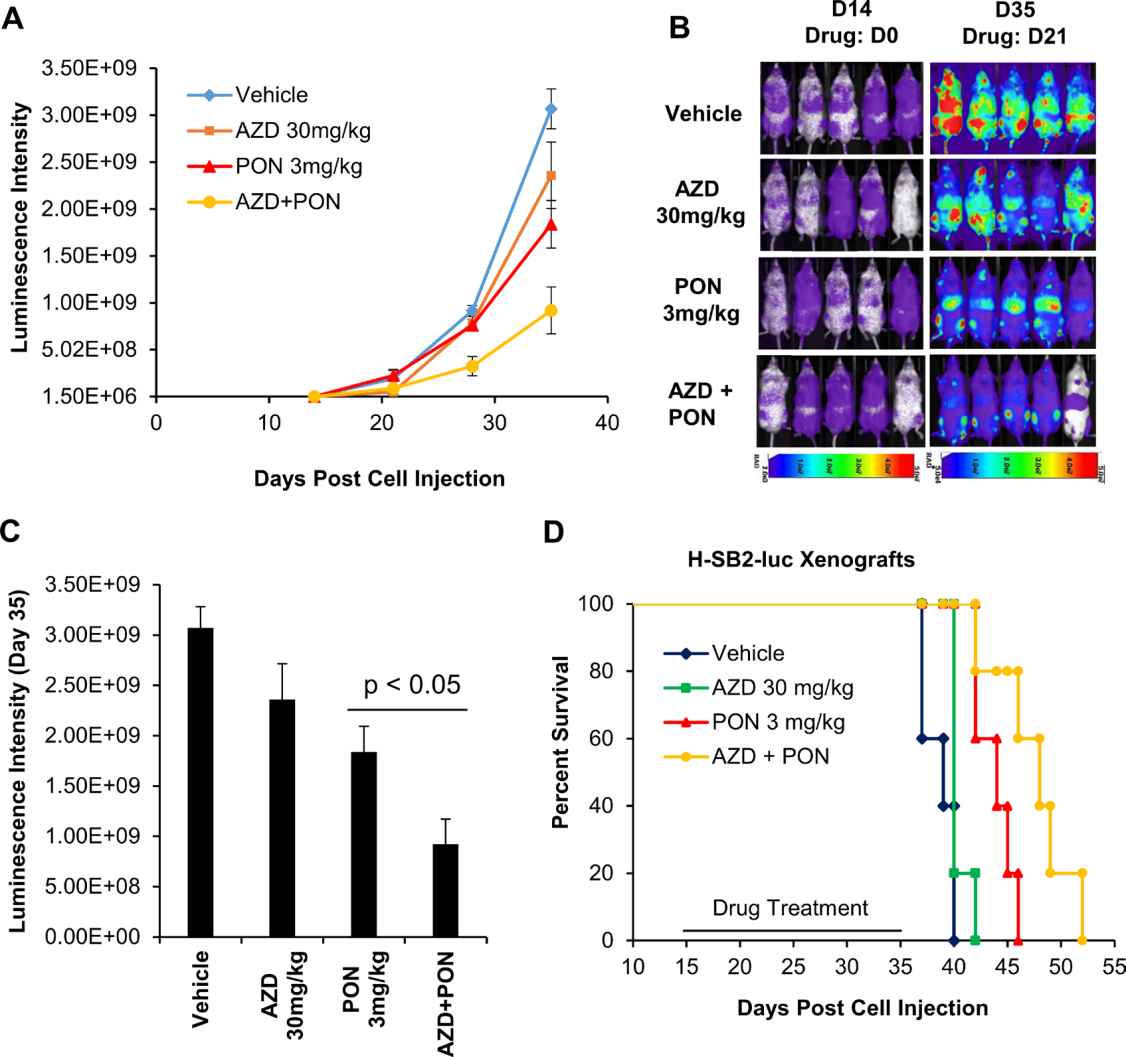
AZD1208 (AZD) and Ponatinib [5] combination treatment improves the survival of mice engrafted with human T-ALL cells (**A**) Twenty sublethally irradiated NSG mice were injected via the tail vein with H-SB2-luc cells (200,000 cells/100 μL PBS). On day 14 mice were divided into 4 groups and treated by oral gavage for 3 weeks daily with vehicle, AZD 30 mg/kg, PON 3 mg/kg, or combination and then the treatment was discontinued and the mice were observed. Tumor growth was monitored by bioluminescence imaging. Luminescence intensities were quantified by using AMIView software. (**B** and **C**) Leukemia burden was assessed by bioluminescence imaging on day 14 and 35, and luminescence intensities from day 35 were quantified from four treatment groups. The luminescence intensity for AZD-PON combination versus PON was significantly different (*p*-value < 0.05). The data shown are the average +/− the S.E.M. from five mice per treatment. (**D**) A Kaplan-Meier plot of the mice engrafted with H-SB2-luc cells treated with vehicle, AZD 30 mg/kg, PON 3 mg/kg, or the combination. The median survival for combination (48 days) versus Vehicle (39 days) was highly significant (*p*-value < 0.005). Kaplan- Meier survival graphs were calculated and compared using a log rank test.

To examine whether this combination treatment would prolong the survival of the mice, sub-lethally irradiated NSG mice were injected with H-SB2-luc cells and then observed for 2 weeks to allow the leukemia to expand. The mice were then treated for three weeks with single or combination therapy. From the bioluminescence (day (D) 14, D21, D28, and D35) measurements in these mice, the combination therapy was better able to kill the leukemic cells (AZD+PON versus PON; *p* < 0.05; Figure 7A–7C). After three weeks, treatment was discontinued and the mice were sacrificed when they experienced significant loss of weight or paralysis (in accordance with the approved IACUC protocol). Median survival was prolonged in mice that received the combination treatment (48 days) compared with vehicle treatment (39 days; Figure 7D). This difference in median survival was highly significant with a *p*-value < 0.005. Thus, combination therapy inhibited the growth of leukemia and prolonged the life of these animals.

## DISCUSSION

In this study, we have demonstrated that pan-PIM inhibitors can block the growth of human T-ALL cell lines. Importantly, the pan-PIM inhibitor sensitive T-ALL cell lines had significantly elevated levels of PIM1 whereas those that were insensitive to these inhibitors do not express PIM1. Other characteristics of the sensitive cell lines were an activated JAK/STAT pathway and lower levels of MYC, whereas the insensitive cell lines bear NOTCH mutations, have elevated levels of phosphorylated and activated AKT, and higher levels of c-MYC. Although the immunophenotype of all three insensitive cell lines corresponded to more mature T-ALL (CD4/CD8 double positive), two of the three sensitive cell lines (H-SB2 and DU.528) showed an ETP/immature (CD4/CD8 double negative) phenotype. Notably, a “mixed” phenotype was evident in the KOPT-K1 cell line, which expressed high levels of PIM1 and evidence of an activated JAK/STAT pathway but also has a mutant NOTCH and expressed a more mature T-ALL immunophenotype. These phenotypes were consistent with the results of our gene profiling analysis of a large T-ALL patient data set, GSE28703 [34], showing that overexpression of PIM1 occurs more commonly in ETP–ALL (75%) but is also found in non-ETP ALL (13%) patients. The existence of subset of patients with a “mixed” phenotype also is suggested by deep sequencing of genetic mutations in ETP-ALL, which found a significant number of patients with a more typical T-ALL phenotype [34, 59]. Our gene profiling analysis identified a 58-gene signature that differed significantly between PIM inhibitor-sensitive versus -insensitive cell lines; this signature overlapped with the differential gene expression identified in ETP versus non-ETP ALL patients. Importantly, the identification of this gene signature suggests that increased PIM1 expression is associated with a network of events that could affect T-ALL cell biology.

Our data also suggested an inverse relationship between the levels of activated NOTCH and the PIM pathways. This relationship between signaling pathways was evident in the genotyping and Cytoscape analysis of T-ALL patient samples. We also demonstrated an inverse relationship between these protein pathways using derived “persister” cells that are resistant to GSI and have an inhibited NOTCH pathway. In these cells, increased levels of PIM1 kinase are seen along with acquisition of sensitivity to the growth suppression by PIM inhibitors. These results are consistent with previous findings: PIM kinases were upregulated in MOLT4 cells upon GSI treatment and NOTCH inhibition [GDS2794, 30]. PIM1 was one of the genes associated with top-ranked BRD4 peaks in two persister (DND-41 and KOPT-K1) cell lines compared to the respective naïve cells (32, Supplementary Table 3). It is possible that PIM1 expression is driven by a superenhancer in these cells. On the other hand, we also observed increased HIF2A and RUNX2 levels in SUP-T1 persister cells, suggesting an activation of STAT signaling, which in turn regulates PIM1 expression.

We have shown previously that inhibition of the PIM kinase pathway is associated with increased ERK1/2 phosphorylation and the combination of PIM and MEK inhibitor acts in a highly synergistic fashion to kill pre-T-lymphoblastic leukemia cells, suggesting the importance of activation of the ERK pathway [17]. Likewise, we found in this study that treatment with pan-PIM inhibitors elevates ERK phosphorylation in sensitive T-All cell lines. mTOR inhibition by rapamycin has been demonstrated to increase ERK activation, possibly through a feedback loop dependent on the S6K-PI3K-Ras pathway [60]. PIM inhibitors may function in a similar fashion since they regulate mTOR activity. Additionally, we have shown that inhibition of PIM kinases increases STAT5 phosphorylation. Since the JAK/STAT pathway regulates PIM levels, this increase in phosphorylation reflects a feedback mechanism that may be driven either by changes in cell surface receptors or hormones that regulate this pathway. Alternatively, PIM inhibitor treatment could lead to a decrease in the phosphatases or other proteins that regulate STAT phosphorylation [61]. To block these pathways and the SRC kinases, e.g., LCK, we combined PIM inhibitors with ponatinib [50] or dasatinib [53]. These TKIs have the potential to block the activity of multiple mutated signaling proteins JAK1, JAK3, IL7R, and FLT3 that have been identified in ETP-ALL [34]. Results of both anti-phosphotyrosine immunoblotting and the examination of downstream signaling proteins, e.g. SRC, suggest that combining PIM inhibitors with ponatinib synergistically decreases tyrosine phosphorylation. Our previous studies demonstrated that PIM inhibitors decrease the protein levels of multiple cell surface receptor tyrosine kinases [54] by regulating protein synthesis.

The *in vivo* animal experiments demonstrate a high degree of synergism between these agents without any significant side effects. Twenty-one days of dual therapy markedly abrogated leukemia as evidenced by optical scanning for luciferase producing cells, smaller spleens (data not shown), and reduced numbers of leukemic cells (based on hCD45 staining) in the peripheral blood and bone marrow. The combination therapy significantly prolonged the life of the treated mice.

Data from human clinical trials demonstrates that serious adverse events have occurred in patients treated with ponatinib, including heart attacks, congestive heart failure, and narrowing of the large arteries of the brain, limiting the use of this agent and preventing further dose escalation [62]. By combining ponatinib with a PIM inhibitor, we have been able to decrease the dose of ponatinib to 1/10th the maximal dose previously used in animal studies.

Whole-genome/transcriptome sequencing and gene expression profiling of chemoresistant high-risk subtypes of pediatric ALL, including ETP-ALL have revealed activating gene fusions, and mutations that could result in constitutively activated tyrosine kinases [34]. Previous genomic studies also suggested that JAK/STAT pathway hyper activation may be a hallmark of ETP-ALL [34]. Recent work confirmed significant activation of JAK/STAT signaling in ETP-ALL patient- derived xenografts relative to non-ETP ALL [63]. Since PIM kinase levels are regulated by the JAK/STAT pathway [64], we believe that the combination of TKI (Ponatinib) and PIM inhibitor (AZD1208/LGB321) will be effective in high-risk pediatric ALL cases, like ETP-ALL.

In summary, our work utilizing T-ALL model cell lines and bioinformatics analysis of clinical datasets demonstrates an important role for PIM kinase activity in T-ALL and especially in ETP-ALL. Tumor xenograft experiments provide strong preclinical rationale for a novel treatment strategy of combining PIM and tyrosine kinase inhibitors for treatment of patients with PIM overexpressing T-ALL.

## MATERIALS AND METHODS

### Human T-ALL cell lines and cell culture

The T-ALL cell lines H-SB2, DU.528, KOPT-K1, CUTLL1, HPB-ALL, and SUP-T1 were cultured in RPMI-1640 supplemented with 2 mM Glutamax (Life Technologies), 10% fetal bovine serum (BioAbChem) at 37^°^C under 5% CO_2_. All cell lines were tested for Mycoplasma.

### Cell viability (XTT) assay

For drug cytotoxicity experiments, T-ALL cells were seeded into 96-well plates at a density of 20,000 cells per well, and pa-PIM kinase inhibitors (AZD1208 & LGB321), Ponatinib or combinations were added at a range of doses for 72 h, using DMSO as control. Cell viability was measured using XTT cell proliferation assay (Trevigen Cat # 4891-025-K) following manufacturer's protocol. Briefly, XTT reagent was added to cell culture (1:2 dilution) and incubated for 4 h at 37°C and 5% CO_2_. The absorbance of the colored formazan product was measured at 450 nm.

### Protein synthesis assay using Click-iT^®^ HPG alexa fluor^®^ 488

At the end of each treatment, cells were moved to L-methionine-free medium containing L-homo propargyl glycine (HPG) for 2 h. A methionine analog HPG is incorporated into proteins during active protein synthesis. This assay is fast, sensitive, non-toxic, and non-radioactive method for the detection of nascent protein synthesis. After treatment, medium was removed followed by cell fixation and permeabilization. Then HPG signaling was detected according to manufacturer's protocol. Detection of the incorporated amino acid utilizes a chemoselective ligation or click reaction between an azide and alkyne, where the alkyne-modified protein is detected with Alexa Fluor^®^ 488 (Catalog # C10428).

### Western blot analysis

At the end of each experiment, cells were lysed in RIPA buffer (Cell Signaling Cat# 9806S). Complete protease/phosphatase inhibitor cocktail (Cell Signaling Cat# 5872S) was added to lysis buffer before use. Protein concentration was determined by Bio-Rad DC protein assay (Bio-Rad). The lysates were then clarified by centrifugation (15,000 g, 10 min) and the resulting supernatant used for immunoblotting. Whole cell lysates were mixed with Laemmli sample buffer and boiled. Aliquots containing equal amounts of protein (30–40 μg) were subjected to SDS-PAGE. Subsequently, proteins were transferred to nitrocellulose membranes and the membrane was blocked by incubation with 5% milk in TBS-T buffer (50 mM Tris-HCl, pH 7.4, 150 mM NaCl, 0.05% Tween 20), for 1 hour at room temperature. The membrane was then incubated overnight at 4°C with the indicated primary antibody in 3% BSA in TBS-T buffer, washed three times in the same buffer and incubated for 1.5 hour with HRP-conjugated secondary antibody. The membrane was then washed three times with TBS-T buffer and visualized by enhanced chemiluminescence (ECL) Western blotting kit according to the manufacturer's instructions (GE Lifesciences, Piscataway, NJ).

### Bioinformatics analysis of T-ALL patients samples from public datasets

T-ALL patient sample expression datasets were analyzed for PIM expression in disease subtypes and gene-profiling analysis was done for these cohorts. The classification of T-ALL samples in cohorts was taken as provided for each dataset analyzed. For cut-off values, gene alterations with significant adjusted *p*-value of ≤ 0.05 and fold change greater than 2 were considered statistically significant. The analysis was carried out using Bioconductor modules and R statistical tools. The LIMMA module from Bioconductor was used for analysis of variance to estimate differential gene expression between the ETP (*n* = 9) and non-ETP (*n* = 35) samples in GSE28703. LIMMA analysis provides an empirical Bayesian method to improve variance estimation and corrects for multiple hypothesis testing by the Benjamini Hochburg false discovery rate method. Gene alterations with significant adjusted *p*-value of ≤ 0.05 or B value ≥ 3 were considered statistically significant. PIM expressions and differential analysis of T-ALL groups were also independently carried out in GSE2156 and Array Express E-MEXP-313. Heatmaps and boxplots were generated using gplots package. Correlations and matrix plots were done using R correlation packages. Bioconductor ReactomePA package and GSEA based Molecular Signature Database (MSigDB) was used to search for significantly enriched candidate pathways in the dataset. Graphical network showing gene-pathway relationships was generated using Cytoscape.

### Affymetrix gene chip expression analysis

Total RNA was extracted from six T-ALL cells using the RNAeasy kit following manufacturer's instructions (QIAGEN Cat #74104). The Genomics Facility Core at University of Arizona Cancer Center performed quality control using the Agilent Bioanalyzer 2100 to confirm all RNA samples had RNA Integrity Numbers (RINs) greater than seven, and quantitate concentration. From the RNA, the Genomics Core produced labeled DNA target using the WT PLUS reagent kit and hybridized it to the Affymetrix^®^ HTA 2.0 Array overnight according to the manufacturer's instructions. Arrays were washed and scanned with the GeneChip Hybridization, Wash, and stain kit and an Affymetrix^®^ Scanner 3000 following manufacturer's instructions. The Affymetrix^®^ Transcriptome Analysis Console v3.0 software was used to analyze resulting data file to identify differentially expressed genes between PIM inhibitor sensitive cells (H-SB2, DU.528, and KOPT-K1) and PIM inhibitor insensitive cells (CUTLL1, HPB-ALL, and SUP-T1) and generated a heat map of differentially expressed genes with the following criteria: Fold Change (linear) < - 3 or Fold Change (linear) > + 3 and ANOVA *p*-value (Condition pair) < 0.05.

### *In vivo* study of pan-PIM inhibitor and Ponatinib combination treatment in mice engrafted with T-ALL

All *in vivo* studies were approved by, and conducted in accordance with the guidelines of the Institutional Animal Care and Use Committees at the University of Arizona Cancer Center. Luciferase-labeled H-SB2 cells were intravenously injected (2 × 10^5^ cell/100 μL) into twenty female NOD/SCID IL-2γ −/− (NSG) mice (5–6 week old) that had been previously irradiated with 2.5 Gy. Three days post injection mice were randomly divided into 4 treatment groups of 5, and treated with Vehicle (Cremophore/Ethanol/PBS – 24/6/70 ratio), AZD1208 30 mg/kg (# A13203, AdooQ BioScience), Ponatinib 3 mg/kg (provided by ARIAD), and AZD1208-Ponatinib combination. These treatments were administered by oral gavage as previously described (44, 54). Starting on day 3, mice were treated daily for 3 weeks. Bioluminescence imaging (BLI) was performed to monitor tumor burden on weekly basis. Briefly, mice were anaesthetized and injected intraperitoneally with 150 mg/kg D-Luciferin potassium salt (Gold BioTechnology, St Loius, MO) solution and anaesthetized by inhalation of 2% isoflurane. Mice were then imaged noninvasively using Spectral Lago X Bioluminescent/Fluorescent Imaging System (Spectral Imaging Instruments, Tucson, AZ). Luminescent activity was quantified using AMIView software. At the end of the study, mice from each group were sacrificed by CO_2_ asphyxiation. Spleen, bone-marrow (BM), and peripheral blood were collected for further analysis. Leukemia burden was also assessed by staining peripheral blood and BM cells with FITC conjugated anti-human CD45 antibody (BioLegend # 304006) and IgG (Isotype control, BioLegend # 400107) and analyzing by flow cytometry. A separate *in vivo* experiment was conducted for percentage survival. Following engraftment of the H-SB2-luc cells mice were monitored for 2 weeks to allow the leukemia to expand. The mice were then treated for three weeks with single or combination therapy and then discontinued. The survival of mice is represented by a Kaplan–Meier plot.

### Statistics

Values reported and shown in graphical displays are the mean +/− standard deviation (S.D.) or standard error of the mean (SEM), as indicated. Comparisons of mean expression across groups were made using an unpaired 2-tailed Student's *t* test. For all comparisons, *P* values less than 0.05 were considered statistically significant.

## SUPPLEMENTARY MATERIALS FIGURES AND TABLES



## Abbreviations

AZD: AZD1208
DAS: Dasatinib
ETP: Early T-cell precursor
GSI: Gamma secretase inhibitor
LCK: Lymphocyte specific tyrosine kinase
LGB: LGB321
PIM: Proviral Insertion in Murine
PON: Ponatinib
T-ALL: T-cell acute lymphoblastic leukemia.
