# Supplementary material for: Targeting the PIM protein kinases for the treatment of a T-cell acute lymphoblastic leukemia subset
Source: Oncotarget. 2017 Mar 17;8(18):30199–216. doi: 10.18632/oncotarget.16320 (PMC5444737; doi:10.18632/oncotarget.16320)
Supplement: Supplementary file 1 [file oncotarget-08-30199-s001.pdf]

Targeting the PIM protein kinases for the treatment of a T-cell acute lymphoblastic leukemia subset

Supplementary Materials

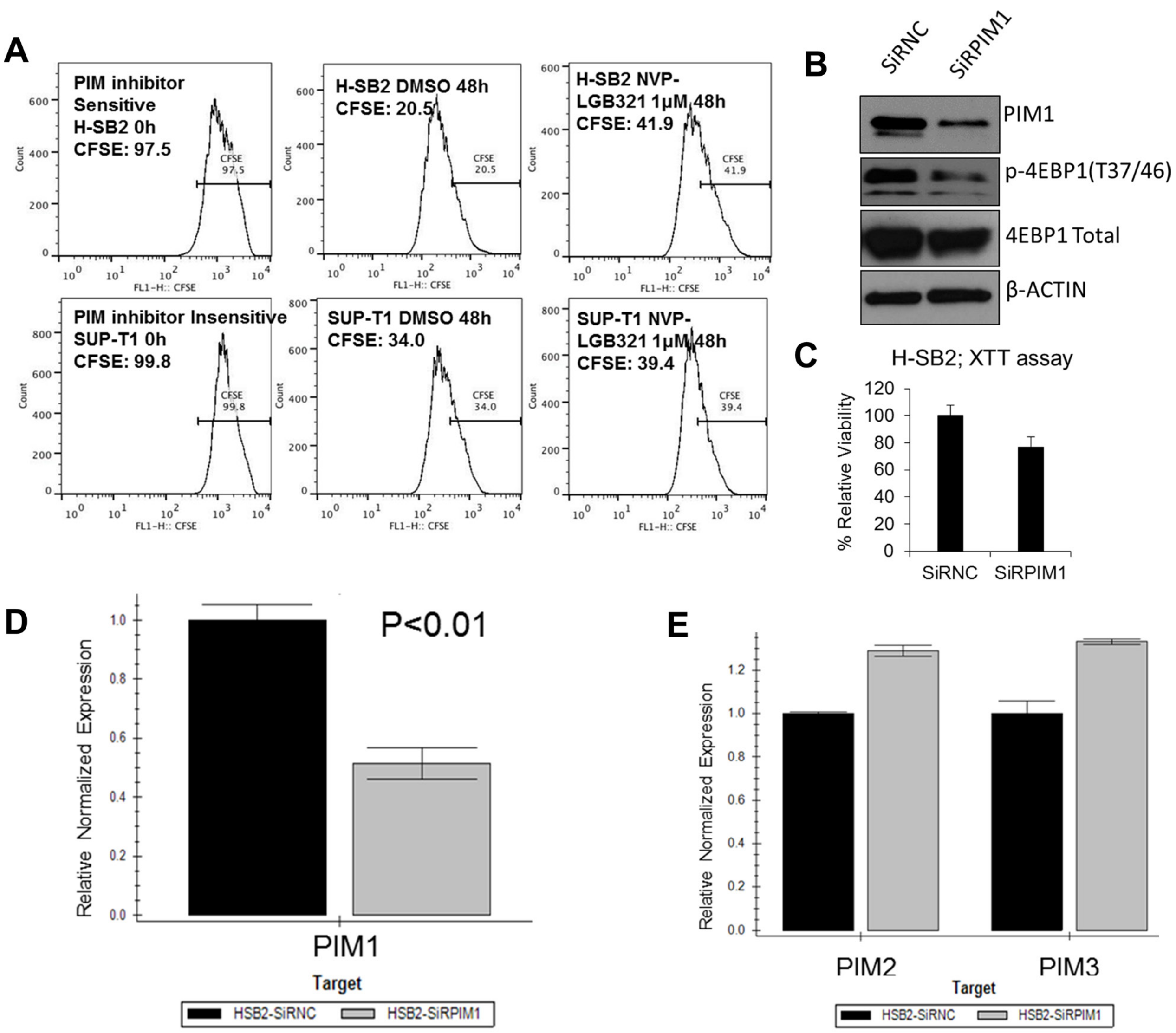

Supplementary Figure 1: Effect of PIM inhibition on T-ALL cell lines. (**A**) H-SB2 and SUP-T1 cells were stained with CFSE and incubated for 48 h with LGB-321 or DMSO. CFSE fractions at 0 h and 48 h were quantified using flow cytometry analysis. (**B–E**) H-SB2 cells were incubated with SiRNC and SiRPIM1 (1 µM for 72 h): PIM1 knockdown efficiency was measured by Western-blot (B) qRT-PCR, quantitative real-time polymerase chain reaction (D) and compared with the Non-silencing Control (SiRNC). (C) Percent cell viability was determined by XTT assay. The growth of SiRNC cells is considered 100% and percentage cell growth for individual treatment is reported relative to the SiRNC cells. (E) PIM2 and PIM3 mRNA expression was analyzed by qRT-PCR

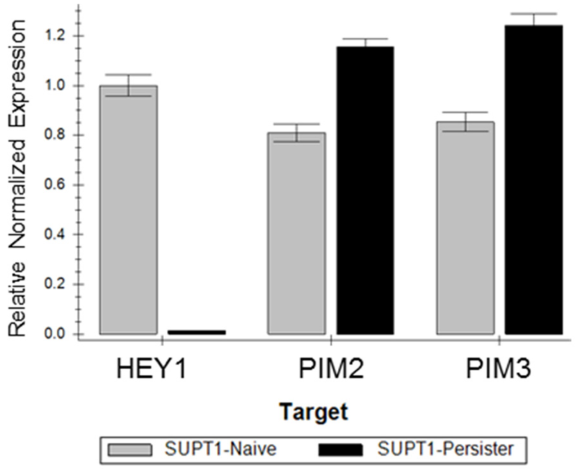

Supplementary Figure 2: Expression of PIM kinases in SUP-T1 naïve and persister cells. Total RNA extracted from SUP-T1 naïve, and pers ister cells and the relative mRNA expression of PIM2 and PIM3 genes were analyzed by qRT-PCR.

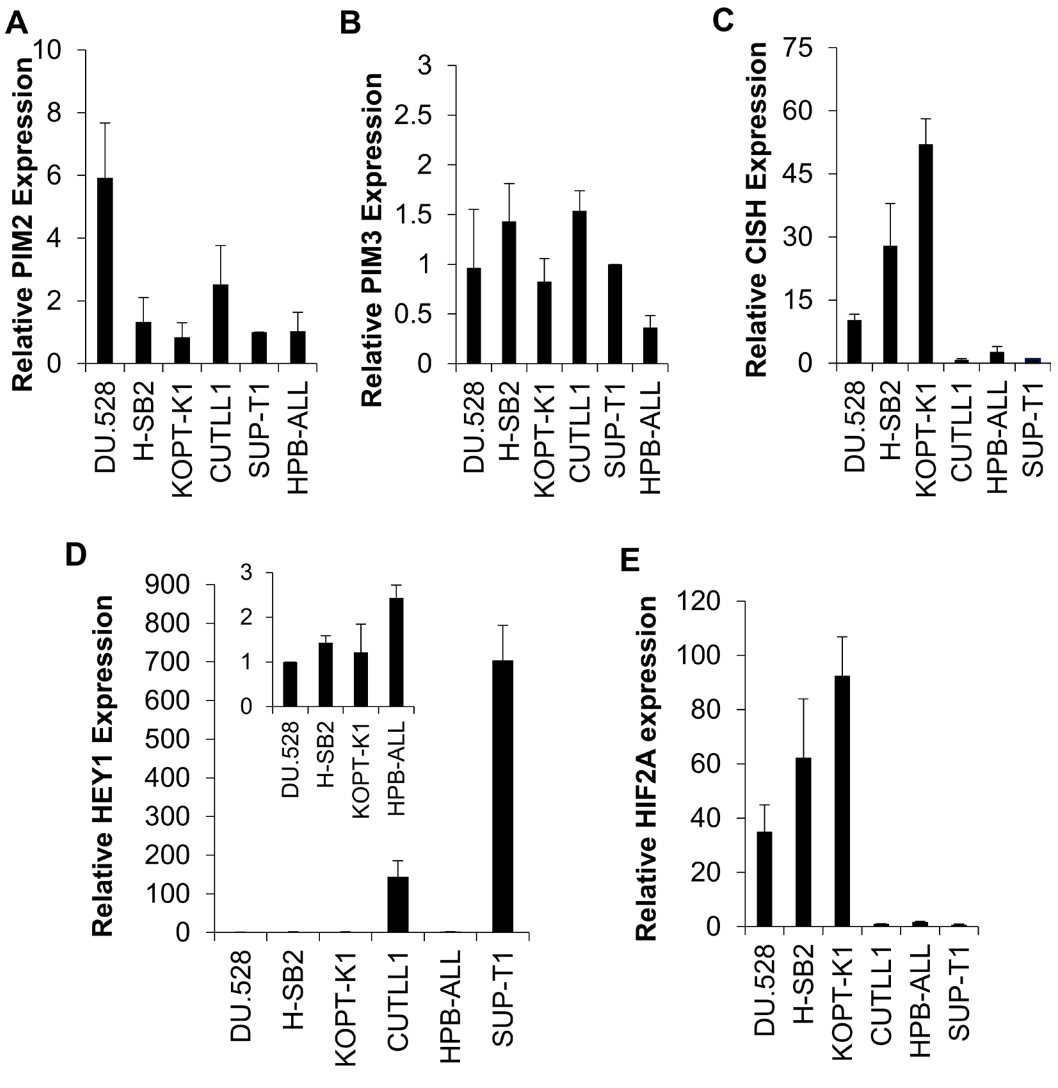

F

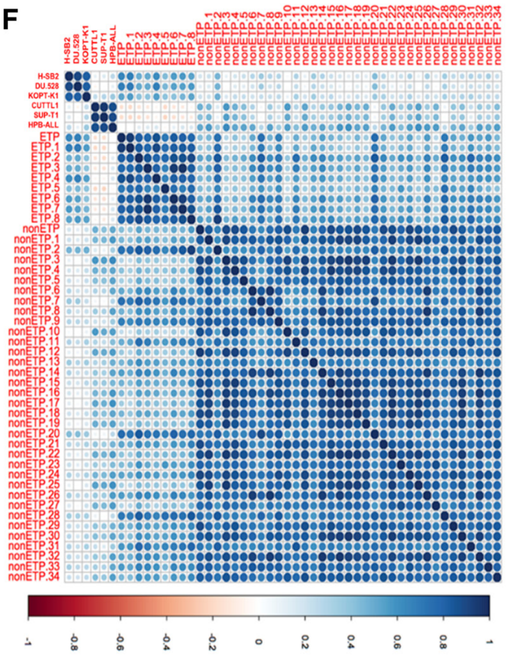

G

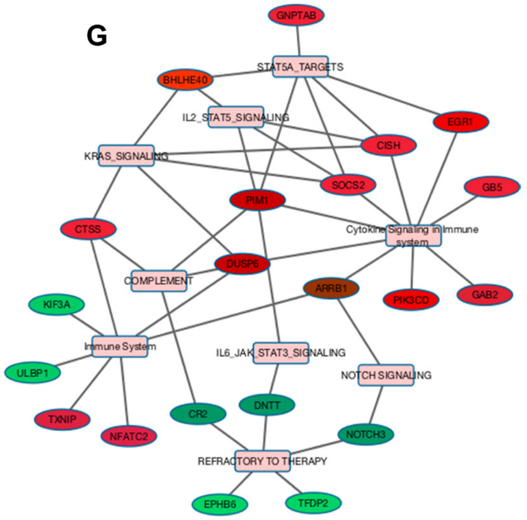

Supplementary Figure 3: Validation of microarray data using quantitative real time-polymerase chain reaction in H-SB2, DU.528, KOPT-K1, CUTLL1, HPB-ALL, and SUP-T1 cell lines. (**A**–**E**) Total RNA extracted from six T-ALL cell lines and the relative mRNA expression of genes shown were analyzed by quantitative real-time polymerase chain reaction. Real-time PCR data shown is the average +/− S.D. of three independent experiments. Correlation analysis of PIM inhibitor-sensitive and insensitive T-ALL cell lines with St. Jude T-ALL patient dataset, GSE28703 (**F**) Correlation analysis of PIM inhibitor sensitive and resistant T-ALL cell lines with patient samples from St. Jude dataset (GSE28703), correlation was done using the R correlation package. (**G**) Network of significantly enriched pathways (Reactome and Msigdb) and their associated genes was constructed using 58-gene signature and Cytoscape. Red circles are the genes in ETP-ALL patients or PIM inhibitor sensitive cells that have increased expression while the green circles are genes that are under expressed.

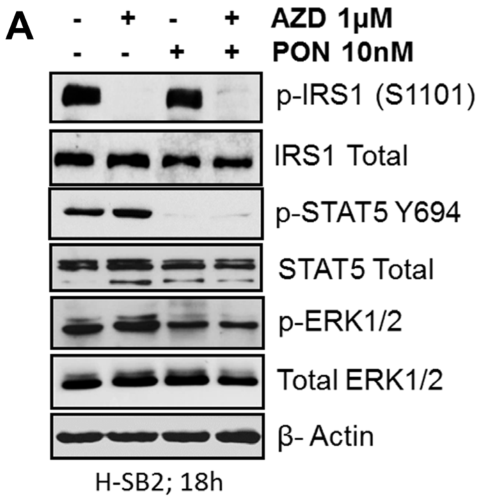

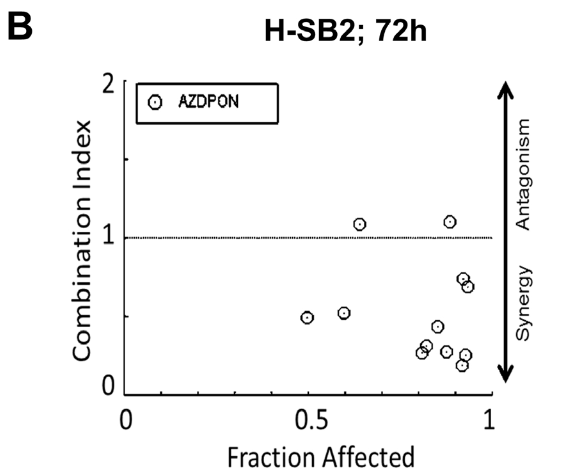

Dose AZD1208	Dose Ponatinib	Effect	Combination Index
0.5 μM	1 nM	0.4992	0.49452
1 μM	1nM	0.59755	0.52245
0.5 μM	5 nM	0.81103	0.27216
1 μM	5 nM	0.82196	0.31306
0.5 μM	10 nM	0.87687	0.27838
1 μM	10 nM	0.91978	0.19135

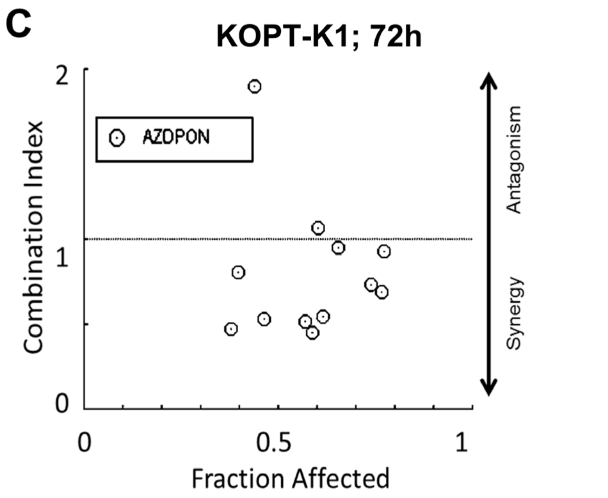

Dose AZD1208	Dose Ponatinib	Effect	Combination Index
0.5 μM	5 nM	0.46592	0.53495
1 μM	5 nM	0.5707	0.51665
0.5 μM	10 nM	0.59028	0.45344
1 μM	10 nM	0.61784	0.54510

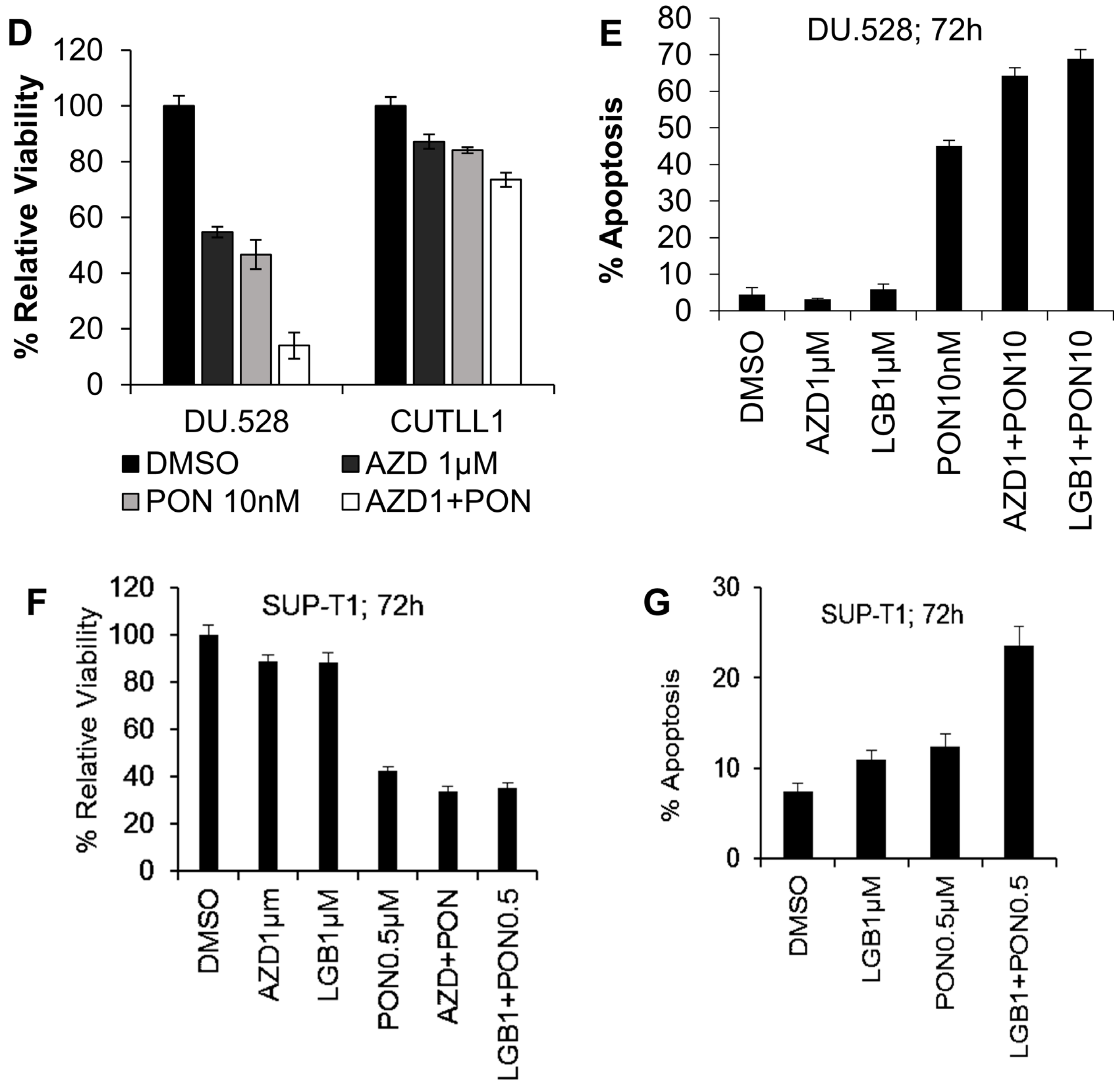

Supplementary Figure 4: Synergistic effect of pan-PIM inhibitor(s) with Ponatinib in a subset of T-ALL cell lines. (**A**) H-SB2 cells were incubated with AZD1208 (1 µM) and Ponatinib (10 nM) for 18 h and cell lysates were immunoblotted with specified antibodies. (**B** and **C**) H-SB2 and KOPT-K1 cells were treated with AZD1208 either alone or in combination with Ponatinib for 72 h and percentage viable cells was determined by XTT assay. The percent growth inhibition of drugs alone and in combination was determined and the combination index (CI) for Ponatinib 5 nM/10 nM and indicated concentrations of AZD1208 combination was determined by Combosyn, Inc. The combination index value of <, = and > 1 indicate synergism, additive effect and antagonism, respectively. (**D**) DU.528 and CUTLL1 cell lines were treated with DMSO, AZD1208 (1µM), either alone or in combination with Ponatinib (10 nM) for 72 h. Percent viability was determined by XTT assay. The growth of DMSO control cells is considered 100% and percentage cell growth for individual treatment is reported relative to the DMSO control cells. (**E**) DU.528 cells treated similar to D, and percentage apoptosis was determined by Guava Nexin assay followed by flow cytometry. (**F** and **G**) SUP-T1 cells were treated with DMSO, AZD1208 (1 µM), LGB-321 (1 µM) either alone or in combination with Ponatinib (500 nM) for 72 h. Percent relative viability was determined by XTT assay. Percentage apoptosis was determined by Guava Nexin assay followed by flow cytometry. XTT and Apoptosis data shown are the average +/− S.D. of three independent experiments.

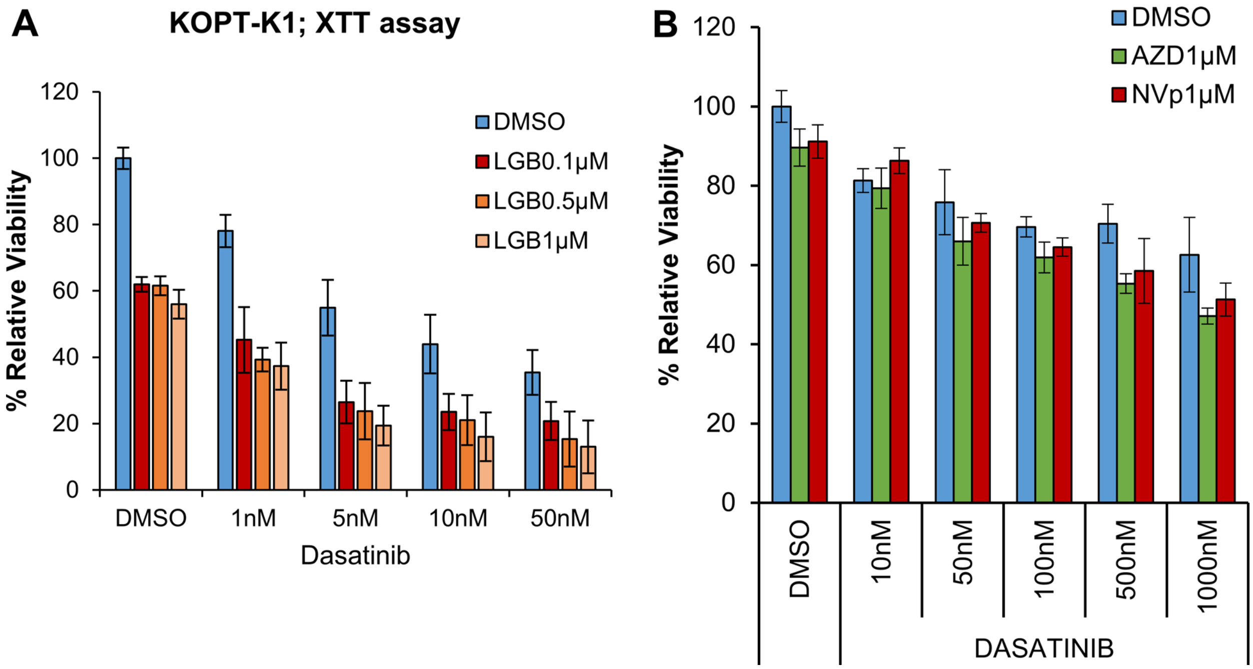

Supplementary Figure 5: Effect of pan-PIM inhibitor(s) in combination with Dasatinib in T-ALL cell lines. (**A**) KOPT-K1 cell lines were treated with indicated concentrations of LGB-321 either alone or in combination with Dasatinib for 72 h and percentage viable cells was determined by XTT assay. (**B**) SUP-T1 cells were treated with indicated concentrations of AZD1208 or NVPLGB-321 either alone or in combination with Dasatinib for 72 h and percentage viable cells was determined by XTT assay. The growth of DMSO control cells is considered 100% and percentage cell growth for individual treatment is reported relative to the DMSO control cells. XTT data shown is the average +/− S.D. of three independent experiments.

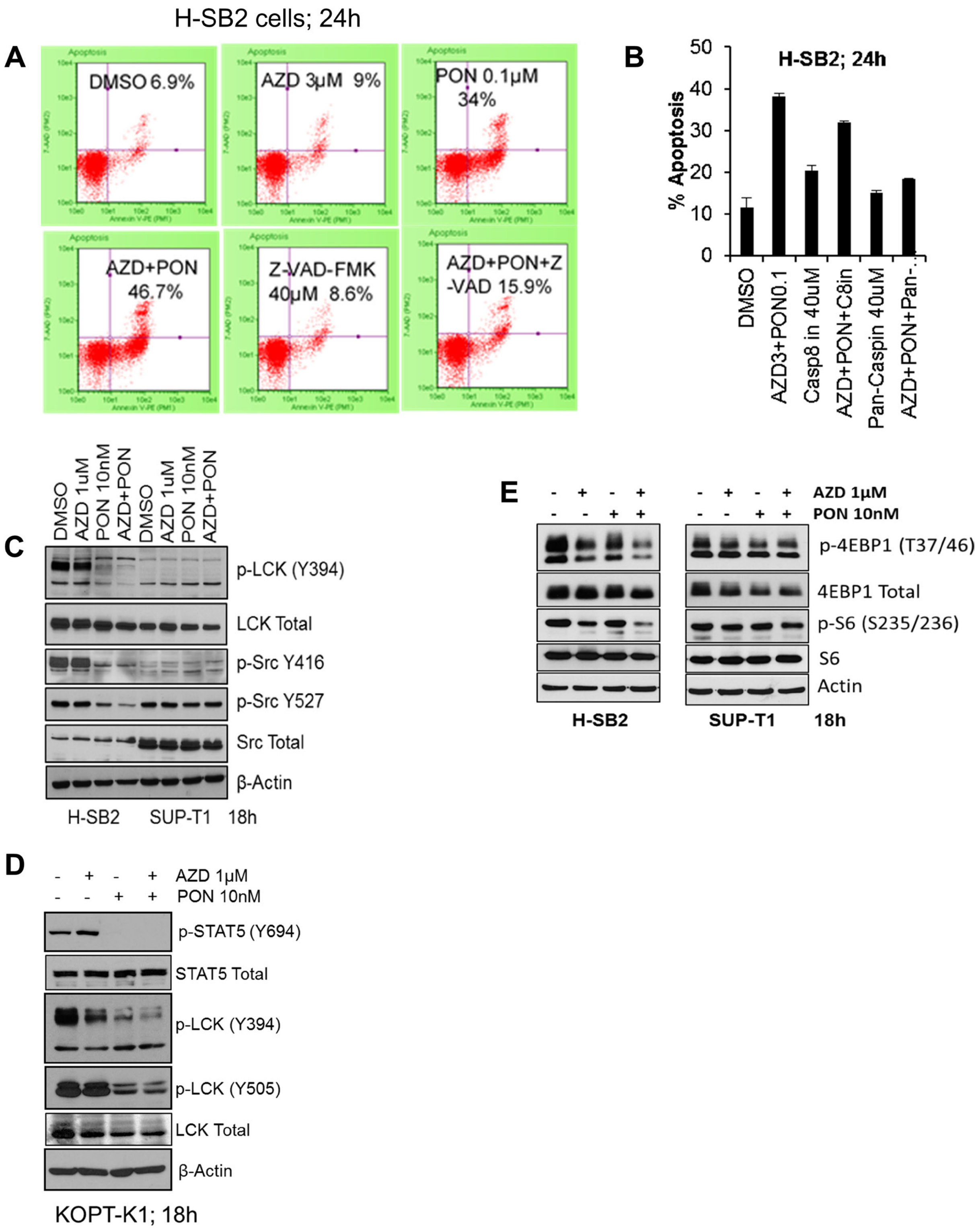

Supplementary Figure 6: Effect of AZD1208 in combination with Ponatinib in T-ALL cell lines. (**A**) H-SB2 cells were pretreated with 40 μM Z-VAD-FMK (pan-Caspase inhibitor) for 2 h, followed by AZD1208 3 μM, Ponatinib 100 nM or combination treatment for 24 h. Percentage apoptosis was analyzed by Guava nexin assay followed by flow cytometry. (**B**) H-SB2 cells were pretreated with 40 μM Z-VAD-FMK or 40 μM Z-IETD-FMK (Caspase-8 inhibitor) for 2 h, followed by AZD1208 3 μM and Ponatinib 100 nM combination treatment for 24 h. Percentage apoptosis was analyzed by Guava nexin assay followed by flow cytometry. Flow cytometry data shown is the average +/− S.D. of three independent experiments. (**C–E**) H-SB2, SUP-T1, and KOPT-K1 cell lines were treated with DMSO, or 1 μM AZD1208 alone or in combination with 10 nM Ponatinib for 18 h and cell lysates were immunoblotted with specified antibodies.

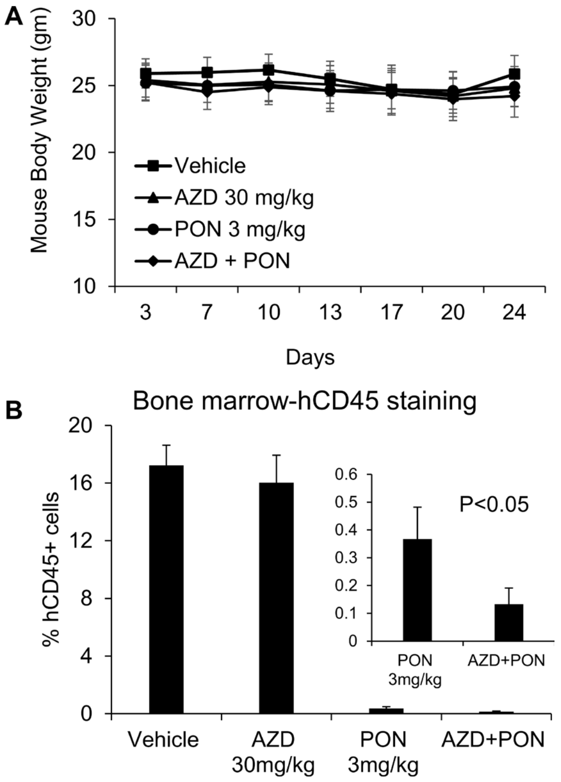

Supplementary Figure 7: AZD1208 and Ponatinib combination treatment in NSG mice engrafted with H-SB2-luc cells. (**A**) Twenty sub-lethally irradiated (2.5 Gy) NSG mice were injected intravenously with H-SB2-luc cells. On day 3 mice were randomly assigned and treated daily with vehicle, AZD1208 (AZD, 30 mg/kg), Ponatinib (PON, 3 mg/kg), or combination by oral gavage for 3 weeks. Body weight of each mouse measured weekly twice to assess the drug toxicity. Data shown are the average +/− S.D. from five mice per treatment. (**B**). Flow cytometric analysis of percent of hCD45+ cells in bone marrow (BM) samples collected from all treatment groups at the necropsy is shown. A significant decrease in the percentage of hCD45+ cells is seen in the BM ($p < 0.005$) harvested from AZD plus PON combination therapy versus PON treatment alone

MATERIALS AND METHODS

Antibodies and reagents

The following antibodies were purchased from Cell Signaling Technology: Anti-PIM1 (Cat#2907), anti-PIM2 (Cat#4730), anti-PIM3 (Cat#4165), anti-AKT (Cat#9272), anti-phospho-AKT (S473, Cat#4058), anti-phospho-4E-BP1 (T37/46) (Cat#2855), anti-phospho-4E-BP1 (S65) (Cat#13443), anti-4E-BP1 (Cat#9452), anti-ERK (Cat#9102), anti-phospho-ERK (Cat#9101), anti-Cleaved Notch1 (Val1744) (Cat#2421), anti-c-MYC (Cat#9402), anti-Caspase 8 (1C12, Cat#9746), anti-Caspase 3 (8G10 Cat #9665), anti-Caspase 9 (C9 Cat#9508), anti-phospho- IRS1 (S1101 Cat#2385), anti-phospho-SRC (Y416 Cat#2101), and anti-phospho-Tyrosine (#9411). Anti-IRS1 (Cat #06-248) antibody was purchased from Merck Millipore. Anti-Bcl-2 (Cat #610539) antibody was purchased from BD Transduction Laboratories. Anti-Bim (Cat#SC11425) antibody was purchased from Santa Cruz laboratories. Anti-phospho-LCK (Y394 Clone#755103), anti-phospho-LCK (Y505 Clone#755216), and anti-LCK (Clone#693010) were purchased from R&D systems. Anti-β-actin (Cat#A3854) was purchased from Sigma. HRP-linked mouse (Cat#NA931V) and rabbit IgG (Cat#NAV934V) were purchased from GE Healthcare Life Sciences. Ponatinib was provided by ARIAD pharmaceuticals. LGB-321 (Cat # A14420) and AZD1208 (Cat # A13203) were purchased from Adooq bioscience. Dasatinib was purchased from Cell Signaling (Cat # 9052). Ruxolitinib was purchased from Selleckchem (Cat # S1378). Caspase 8 inhibitor (Z-IETD-FMK) was purchased from MBL life science (# 4805-510) and pan-caspase inhibitor (Z-VAD-FMK) was purchased from APExBio (# A1902).

Accell siRNA transfection in H-SB2 cells

H-SB2 cell were cultured in the presence of 1 μM Accell siRNA specific for human PIM1 (Cat # A-003923-17) or non-silencing control (Cat # D-001910-01-20) in Accell delivery media (Cat # B-005000-500) according to the manufacturer's instructions (Dharmacon). Cells were analyzed after 72 h in culture.

Cell viability (XTT) assay

For drug cytotoxicity experiments, T-ALL cells were seeded into 96-well plates at a density of 20,000 cells per well, and pa-PIM kinase inhibitors (AZD1208 & LGB-321), Ponatinib or combinations were added at a range of doses for 72 h, using DMSO as control. Cell viability was measured using XTT cell proliferation assay (Trevigen Cat # 4891-025-K) following manufacturer's protocol. Briefly, XTT reagent was added to cell culture (1:2 dilution) and incubated for 4 h at 37°C and 5% CO_2. The absorbance of the colored formazan product was measured at 450 nm.

Development of SUP-T1 persister cells

Persister cells were developed as described previously (24) by repeatedly exposing SUP-T1 cells to a 1 μM of Gamma Secretase Inhibitor, Compound E (EMD4 Biosciences) over the period of 7 weeks, replenishing the inhibitor every 3–4 days. Persister cells were characterized as reported previously.

Real-Time PCR

Total RNA was isolated using RNAeasy kit and 1 ug RNA was used to synthesize cDNA (Bio-Rad, Cat # 1708890) following the manufacturers protocol. The quantification of real-time PCR products was performed using SsoAdvanced™ Universal SYBR® Green Supermix (Bio-RAD, Cat#1725271) on a CFX96 Real-Time System. Samples were assayed in triplicate and the data were normalized to 18S mRNA levels. Primer sequences are provided in TableS4.

Cell cycle analysis

Cell cycle progression was analyzed by Guava Easy Cyte flow cytometer. After drug treatment cells were centrifuged, washed with PBS, and fixed in 70% ethanol solution overnight at −20°C. Fixed cells were centrifuged and pellet was washed with PBS before stained using a propidium iodide-RNase A solution (PBS containing 50 ug/ml Propidium Iodide, 0.1 mg/ml RNase A, and 0.05% Triton X-100) for 30–40 min at 37°C in the dark.

Assessment of apoptosis by annexin V staining

The percentage of apoptotic cells were detected by using Guava Nexin reagent (Millipore #4500-0450) following the manufacturer's instructions using the Guava Easy Cyte flow cytometer. Briefly, cells were mixed with Guava Nexin reagent (1:2 dilution) and incubated for 20 minutes at room temperature in the dark. In the Guava Nexin assay, Annexin V-PE positive stained cells are in the bottom right quadrant of the display and represents early apoptotic cells. While the cells with 7-AAD staining appear in the top right quadrant and represents the late apoptotic and dead cell populations.

CFSE staining

T-ALL cells were stained using cell Trace CFDA SE dye, (Vybrant CFDA SE cell tracer kit, Invitrogen, Cat # V12883) diluted in PBS to give a final staining concentration of 5 μM. The staining reagent containing the cells were mixed well until homogeneity and incubated at room temperature for 10 min. The solution was then centrifuged at 800 rpm for 5 min and the supernatant was discarded. The cells were washed by resuspending the pellet in 1-2 ml of fresh RPMI Medium, centrifuging at

800 rpm for 5 min and discarding the supernatant. After washing, the cells were treated with DMSO or AZD1208 for 48 hours and the percentage CFSE positive cells quantitated by flow cytometry.

Generation of the luciferase-expressing, H-SB2 cells

T-ALL cells line, HSB2 was electroporated with plasmid (Pluc-ff-Zeo), using Human CD34+ cell Nucleofector kit and Nucleofector II Device (Amaxa scientific, Allendale, NJ). Positive cells were selected by gradually increasing concentrations of Zeocin. Luciferase activity of selected cells were tested by Dual-Glo luciferase kit (Promega, Madison, WI).

Supplementary Table 1: Imuunophenotype of 6 human T-ALL cell lines and ETP-ALL

	T-/NK cell	Non-lineage /Stem cell
DU.528	$CD1^-$, $CD3^-$, $CD4^-$, $CD8^-$	$CD34^+$, $CD71^+$, TdT^-
H-SB2	$CD1^-$, $CD3^-$, $CD4^-$, $CD8^-$	$CD34^{+/-}$ $CD38^+$ $CD71^+$, TdT^-
KOPT-K1	$CD1a^+$, $CD3^+$, $CD4^+$, $CD8^+$	$CD34^-$, $CD38^+$, $CD71^+$, TdT^+
ETP-ALL	$CD1a^-$, $sCD3^-$, $CD4^-$, $CD8^-$	$CD34^+$, TdT^-
CUTLL1	$CD1a^+$, $CD3^+$, $CD4^+$, $CD8^+$	$CD34^-$, TdT^+
SUP-T1	$CD1^+$, $CD3^+$, $CD4^+$, $CD8^+$	$CD38^+$, $CD71^-$, TdT^+
HPB-ALL	$CD1^+$, $CD3^+$, $CD4^+$, $CD8^{+/-}$	$CD34^-$, $CD38^+$, TdT^+

[1, 4, 29, 30].

Supplementary Table 2: List of top 135 coding and non-coding genes that significantly differentiate (Fold Change, linear < − 3 or > + 3 and ANOVA *p*-value (Condition pair) < 0.05) PIM inhibitor sensitive cells (H-SB2, DU.528, and KOPT-K1) from PIM inhibitor insensitive cells (CUTLL1, SUP-T1, and HPB-ALL). See Supplementary_Table_2

Supplementary Table 3: Gene set enrichment analysis based, the molecular signatures database search: Significantly enriched pathways and list of genes

GSEA Genesets	Genes	*p*-value	FDR *q*-value
CHIARETTI T-ALL Refractory to chemotherapy	NOTCH3, TFDP2, DNTT, CR2, EPHB6	3.11E-10	1.49E-06
WIERENGA STAT5A targets	EGR, SOCS2,GNPTAB, BHLHE40, PIM1, CISH	2.76E-07	1.46E-04
COMPLEMENT	PIM1, CTSS, DUSP6, CR2	1.07E-04	1.78E-03
IL2-STAT5 signaling	PIM1,CISH, SOCS2, BHLHE40	1.07E-04	1.78E-03
KRAS signaling	CTSS, DUSP6, CISH, SOCS2, BHLHE40	1.07E-04	1.78E-03
IL6-JAK-STAT3 signaling	PIM1, DNTT	5.11E-03	3.19E-02
NOTCH signaling	ARRB1, NOTCH3	7.07E-04	7.40E-03

False discovery rate (FDR) *q* value of 0.05 was used as cutoff.

Supplementary Table 4: List of quantitative real-time PCR primer sequences

Gene (Human)	Sequence
PIM1	Forward 5′-CGACATCAAGGACGAAAACATC-3′<br>Reverse 5′-ACTCTGGAGGGCTATACACTC-3′
PIM2	Forward, 5′-GAACATCCTGATAGACCTACGC -3′<br>Reverse, 5′-CATGGTACTGGTGTCGAGAG -3′
PIM3	Forward, 5′-GACATCCCCTTCGAGCAG-3′<br>Reverse, 5′-ATGGGCCGCAATCTGATC-3′
CISH	Forward, 5′-AGAGGAGAGAAATACACGTC-3′<br>Reverse, 5′-TTACACAACTGAAAATCGGC-3′
HEY1	Forward, 5′-CGACGAGACCGGATCAATAAC-3′<br>Reverse, 5′-AGCAGATCCTTGCTCCATTAC-3′
C-MYC	Forward, 5′- TTGAGGAGTGCGAGGTGCTG -3′<br>Reverse, 5′- ATCTGGGCCAGCAGAAGTGC -3′
HIF2A	Forward, 5′- CCCATGTCTCCACCTTCAAG -3′<br>Reverse, 5′- GGCTTGCTCTTCATACTCCAG -3′
ID1	Forward, 5′-TTGGAGCTGAACTCGGAATC-3′<br>Reverse, 5′-ACACAAGATGCGATCGTC-3′
18S	Forward, 5′-GTAACCCGTTGAACCCCATT-3′<br>Reverse, 5′-CCATCCAATCGGTAGTAGCG-3′
